# Feature determination from powered wheelchair user joystick input characteristics for adapting driving assistance

**DOI:** 10.12688/wellcomeopenres.12280.3

**Published:** 2018-05-10

**Authors:** Michael Gillham, Matthew Pepper, Steve Kelly, Gareth Howells

**Affiliations:** 1 Department of Engineering and Digital Arts, Jennison Building, University of Kent, Canterbury, Kent, UK

**Keywords:** assistive technology, powered wheelchair, rehabilitation

## Abstract

**Background**: Many powered wheelchair users find their medical condition and their ability to drive the wheelchair will change over time. In order to maintain their independent mobility, the powered chair will require adjustment over time to suit the user's needs, thus regular input from healthcare professionals is required. These limited resources can result in the user having to wait weeks for appointments, resulting in the user losing independent mobility, consequently affecting their quality of life and that of their family and carers. In order to provide an adaptive assistive driving system, a range of features need to be identified which are suitable for initial system setup and can automatically provide data for re-calibration over the long term.

**Methods**: A questionnaire was designed to collect information from powered wheelchair users with regard to their symptoms and how they changed over time. Another group of volunteer participants were asked to drive a test platform and complete a course which represented manoeuvring in a very confined space as quickly as possible. Two of those participants were also monitored over a longer period in their normal home daily environment. Features, thought to be suitable, were examined using pattern recognition classifiers to determine their suitability for identifying the changing user input over time.

**Results**: The results are not designed to provide absolute insight into the individual user behaviour, as no ground truth of their ability has been determined, they do nevertheless demonstrate the utility of the measured features to provide evidence of the users’ changing ability over time whilst driving a powered wheelchair.

**Conclusions**: Determining the driving features and adjustable elements provides the initial step towards developing an adaptable assistive technology for the user when the ground truths of the individual and their machine have been learned by a smart pattern recognition system.

## Introduction

There is no typical powered wheelchair (PWC) user. Individuals may be suffering from neurological trauma or disease, or be affected by musculoskeletal trauma or disease; and they may be of any age. Increasingly, people are living longer and therefore may require mobility assistance for much longer. One publication in 2010 ^1^ investigated these issues; the research concluded that the literature was lacking in regard to the PWC user quality of life and their ability to self-maintain. The Canadian Occupational Performance Measure (COPM), developed by Law *et al*. in 1994 ^2^, measures the users’ perception of their own self-care and living capabilities by using a questionnaire which usually takes around thirty minutes to complete. This methodology has been applied in a lot of research, some for PWC users, according to a review of the methodology ^3^. Mills *et al.* ^4^ propose a conceptual framework, which includes a range of factors likely to affect user performance. They identify 10 tools used to assess the user driving abilities for the purpose of more suitable adjustment of the PWC to the individual user's needs.

Earlier research into young people’s needs, with regards to the benefits of the PWC, had indicated that children who used PWCs had much better spatial awareness and cause-and-effect skills than their peers who did not use PWCs ^5^. However more recent research ^6^ concluded that the benefit to the user was only positive when both the PWC and their environment were of a suitable ‘fit’, and could be quite negative when the child felt excluded from social integration; for example, due to the bulkiness of the PWC restricting their movements in confined environments.

A study of the elder PWC user found that one of the immediate benefits from issue of a PWC was an increased independence and a feeling of well-being ^7^. Furthermore, the research suggested that despite this euphoria many of the elderly users were anxious about driving outdoors, which may be due to a fear of accidents, particularly toppling over, and the issue of a breakdown leaving them stranded. The research noted that this user group were dissatisfied with the wheelchair service, quoting long waiting times and having serious concerns that the chair would not meet their changing needs over time.

There has been much research in the field of providing PWC users with smart and assistive systems ^8, 9^; however, most users do not like having their control taken away from them, essentially disempowering them ^10^ rather than assisting them to overcome challenges that their disabilities present. This essentially means that each individual would need the assistive system to be adapted and adjusted to their individual needs and requirements, and for the system to be re-tuned as their needs change over time. This is a substantial constraint for any manufacturer or developer of technology.

Therefore each person’s needs from any smart assistive PWC is quite unique and specific to them, hence current commercially available assistive technology tends to be specialist equipment, specifically designed and built for each individual, or a group suffering from a particular illness, or alternatively is some standard hardware/software which has been adapted for the particular individual. This means that assistive technology is very costly in both equipment and technical maintenance, particularly when the device may require constant adjustment ^11^.

In order for manufacturers to be able to mass produce devices at an affordable cost, there needs to be a sufficient volume of production. Therefore, there is a need to develop assistive PWC technology which is adaptable to a wide range of users’ clinical needs, whilst also being adaptable to the individual’s personal preferences. This would require a smart system which monitors the user's performance and adjusts the system accordingly; this information may also be directly related to their medical condition, which could potentially provide clinicians with more data to base their diagnosis and subsequent treatments on.

Currently, health authorities across Europe provide adaptive and assistive technology for those who need it on an individual basis; therefore PWCs are adapted to each person, which is expensive and very time consuming. The PWC user is subjected to an ability-to-operate test and, in the UK ^12^, will only be given an NHS funded PWC if they meet this criterion. For those individuals who do not meet the requirements the only alternative is to either have an assistant in constant attendance, or not to use a PWC unless they can buy their own, with the associated re-tuning costs.

According to research ^13^ there are a wide range of diseases which may cause sufficient disabilities to prevent individuals from operating a PWC without assistance, these have been extrapolated from the US population at the time of the research to fit the current (2016) EU population and are listed in Table 1 by diagnosis, with some of the typically associated symptoms. According to our research and experience, whilst developing and testing our driving assistance technology ^14– 16^, providing simple collision avoidance or navigational assistance would not be sufficient to allow unmonitored use of the PWC, due to safety risks. There needs to be a synergetic assistance which adapts the assistance to the needs and requirements of the individual as they change over time, and most importantly keeps the user in full control of the PWC motion at all times.

**Table 1.** Estimated potential smart wheelchairs users, organised by diagnosis, in the EU.

Diagnosis	Prevalence (millions) (Lower–upper)	% who need wheelchair (Lower–upper)	Typical symptoms	% with symptoms (Lower–upper)
Alzheimer disease	3.6–6.4	10–20	Attention, agitation, and impulse control Executive reasoning	45–52 35–45
Amyotrophic lateral sclerosis	0.04–0.05	46–80	Fatigue/weakness Head/neck movement	20–26 20–26
Cerebral palsy	1.1–1.3	80–90	Spasticity Tremor Hemiplegia Ataxia Dystonia Executive reasoning	70–90 10–20 10–20 5–10 15–20 30–40
Multiple sclerosis	0.4–0.6	65–75	Spasticity Tremor Fatigue/weakness Head/neck movement Ataxia Executive reasoning	65–90 5–7 43–90 43–90 23–84 30–70
Parkinson disease	1.4–1.6	5–15	Visual field neglect Tremor Bradykinesia Executive reasoning	85–95 60–65 10–15 25–45
Traumatic brain injury	4.6–5	15–25	Visual field neglect Visual field loss Spasticity Hemiplegia Tremor Bradykinesia Fatigue/weakness Head/neck movement Attention, agitation, and impulse control Executive reasoning	20–80 15–25 35–50 45–50 20–30 20–25 35–50 35–50 20–60 50–60

### Research aims

This research seeks to determine features which would identify the changing needs of PWC users and to distinguish the elements which would be required to make adjustments to an assistive PWC system, which would then be able to adapt to the users’ needs as they change over time. In order to do this we need to identify and quantify the severity of the problem. This will require identifying how often they use their PWC, how many collisions occur and when, what problems they have and how that may relate to their specific disability.

The user joystick input trajectory quality can be used as a measure of the level of assistance required to assist an individual ^17– 19^; however research is sparse with regards to quantifying and qualifying that user input with the intention of providing precise assistance for that individual when so required. We have also identified from our previous research that the user’s approach angle to doorways and their proximity to obstacles when navigating the environment can be a measure of their ability to drive, this research further investigates the suitability of collision avoidance as an identifying feature for feedback to adapt the system to better suit the individual's’ navigation assistance requirement.

The ultimate objective of this research is to provide the adjustable elements which can be used to first improve the user’s own input quality, keeping them in full control for as long as possible and only when necessary moving to the next layer of assistance, which would also provide progressively more collision avoidance assistance, and then to the next level providing steering assistance, and finally to the higher level where the joystick input has now become digital. This methodology we believe will allow individuals who have been precluded from being prescribed a PWC to now be eligible for a smart adaptable assistive PWC, where the assistive system would also be able to step-in and provide the necessary assistance to keep the user in full and safe control of their PWC.

### Powered wheelchair control

The electric powered wheelchair is usually controlled by a joystick which provides a digitised proportional input where one axis provides the turn proportionality and the other axis provides the forward and reverse proportionality. The powered wheelchair platform can be described as a unicycle or a two wheeled non-holonomic tank like mechanism ^14^ which has the following kinematic:

$${\dot{x}}_{body}=v_{body}=\frac{v_{right}+v_{left}}{2}\;(1)$$

$${\dot{\theta }}_{body}=\omega_{body}=\frac{v_{right}-v_{left}}{W}\;(2)$$

Where:

*_right_* and *_left_* are the velocities of the individually driven rear wheels.
*W* is the distance between the rear driving wheels.

This means that the platform motion is restricted by these equations; this means that for a wheel separation of half a metre the platform can rotate 4 times faster than the forward velocity. The joystick input device has a very similar mathematical relationship; however the distance between the two drive wheels would need to be two metres for there to be an equally proportional relationship between the joystick and the motion.

According to one control system manufacturer; they employ a ‘Virtual Restrictor Plate’ (VRP) ^20^ for the purpose of allowing the medical practitioner some degree of freedom to adjust the distance the user is required to move the joystick with regard to the actual motion of the PWC, whilst maintaining a safe ratio of speed to turn. Other methods of modifying the shape have been evaluated ^21^ however the basic principle is still the same, to map or scale in some way the joystick to provide the user with their desired platform motion. There are usually up to five joystick mapping profiles ^20^ which are used for different speed and turn rates such that the input better matches the desired output, akin to changing gear in a motorised vehicle. This means that the user can use the same joystick movement to drive have a much finer control at low speeds in confined environments and or conversely little joystick movement to drive at speed outdoors. This scaling will affect turn speeds, forward and reverse speeds, acceleration and deceleration. The outcome should be that the user has the PWC set up so that they feel safe and competent to drive in restricted and open environments. The Kent and Canterbury hospital wheelchair technicians and prescribing clinicians we interviewed stated that this process may take several sessions and sometimes a satisfactory outcome is not achieved.

A further challenge to meeting user need is that their ability and ease to move the joystick may change significantly over time. In the case of the smart PWC, the system would need to adjust the dynamic parameters in the collision avoidance and the trajectory generation according to the joystick input and the profile selected by the user.

**Figure 1.**
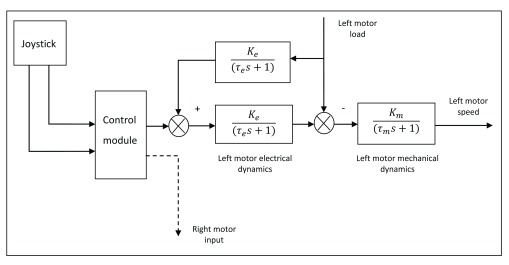
Typical feed-forward PWC control schematic.

The mapping process will not only need to map the position of the joystick to the desired velocity for each motor driven wheel according to Equation 1 and Equation 2 it will also need to provide some time delay ramp to the rate of change of the joystick such that the motor acceleration is smooth and jerk is minimised. The most common method is to use a feed-forward control approach, shown in Figure 1, where certain parameters can be adjusted to suit the needs of each user of the platform whilst remaining within the boundaries of the electrical and mechanical system dynamics ^22^. This process is implemented in the control algorithms developed by the control system manufacturer.

The most common parameters which always require adjustment to the individual for each profile (driving situation) are:

- forward speed range
- forward +/- acceleration
- reverse speed range
- reverse +/- acceleration
- turn speed range
- turn +/- acceleration.

The current method of setting-up a PWC is essentially down to trial-and-error, and the procedure (after discussion with the wheelchair service, dealers, and clinicians) can be as follows:

- Match the power module load compensation to the motor loads to ensure the loading between motors is the same and it is driving without a bias to one side on flat ground.
- Adjust the positive and negative acceleration on turning, forward, reverse, pulling away and stopping.
- Tune for each profile such that the performance is evenly spread across the range of profiles.
- Fine tune each profile with the user to suit their desires and needs.

Some of the observations commonly reported, according to PWC dealers, hospital wheelchair service technicians and clinicians we spoke to, were:

- Motor load compensation can be very difficult to achieve on older chairs where it is not possible to compensate for differences in left and right motor load. Additionally compensation may only be effective at one power level. This means that the chair may drive in a straight line on a vinyl surface, but veer on a carpeted surface. Joystick users can compensate for this change in drive characteristic. However the switch user is not able to do so.
- Aggressive acceleration or too high forward and turning speed can frighten some users and made them reluctant to drive the chair.
- Some users with reduced hand function required reduced joystick throw – that is 50% deflection gives 100% speed.
- Sometimes it is necessary to reverse the polarity of the Forward/Reverse action if the user found it easier to pull the joystick rather than push.
- It is important to keep asking, every time you see the user, if the settings are suitable.
- There are not enough resources for regular visits to the user to check and adjust PWC tuning.
- Users may not report that they are having difficulty driving their chair. Perhaps for fear that the chair will be taken away or because they don’t think that anything can be done to improve the chair setup or because they have given up asking.
- Therefore a smart wheelchair which can identify the user’s driving characteristics and detect from those characteristics whether the chair requires retuning, should be of great benefit to that user.

### Ethical statement

The project was subject to the University’s formal procedure for ethical consideration of projects involving human participation, under the auspices of the Faculty of Sciences’ Research Ethics Advisory Group. Ethical approval was granted by NRES Committee East of England, REC reference: 14/EE/0164 under the title: Evaluation of a Powered Wheelchair with collision avoidance.

## Methods

Whilst there is some guidance on the analysis of driving features ^23^ there remains the need to determine which features are good indicators of the user’s ability to control their PWC safely in their respective environments, especially if their ability changes over time. A potential set of features is given in Table 2. These are based upon the symptoms listed in Table 1 and some adjustable parameters for PWC control systems, and also information on the proximity of obstacles.

**Table 2.** Measurements for a potential feature-set.

**Symptoms**	**Reactions**	**Measurements**
Tremors and involuntary movements of the joystick	Continuous sinusoidal component and sudden motion	• Position • Velocity • Frequency
Attention, tiredness, and general fatigue	Increasingly irregular motion and proximity to obstacles, operational time reduction	• Position • Velocity • Proximity • Time
Muscular stiffness and weakness	Directional bias and amplitude change of muscular activity	• Position • Velocity • Time
Observational and visual bias	Hesitation and preference when driving in certain directions, proximity to obstacles	• Position • Velocity • Proximity • Time
Reasoning, confusion, panic, and agitation	Hesitation when driving, directional changes, stop-start, proximity to obstacles, sudden motion, possible nervous tremors	• Position • Velocity • Frequency • Proximity • Time

A three pronged approach was undertaken in order to investigate the problem. The first approach was to locate and obtain data from PWC users by using a questionnaire ( Supplementary File S1). This was undertaken by attending disability exhibitions and conferences and asking visitors in powered wheelchairs to take part in the data collection. The anonymous questionnaire with a self-addressed envelope was included and out of nearly 70 questionnaires some 11 participants responded (A group), the anonymous data was simply given a random identifier when the envelope was opened and their backgrounds with the associated identifiers are given in Table 3. They were also asked to self-score the number of collisions and the range of their abilities, for one of their self-defined ‘good days’ and for one of their ‘bad days’. Participants were clearly informed that they were partaking in research and that the data would be used solely for research purposes, both verbally and in writing on the questionnaire, and that by returning the questionnaire they would be giving their consent to participate. The returned questionnaire data had no personal identification attached and therefore is fully anonymised. This does not alter or distort the scientific meaning.

**Table 3.** Powered wheelchair user questionnaire background data.

Identifier	Driving ability	Number of years in wheelchair		Reason for PWC	Difficulties
		*Manual*	*Powered*		
**A1**	5	3	6	Physical strength	None at present
**A2**	5	0	20	Physical strength	Slopes and kerbs
**A3**	4	14	6	Guillain–Barré syndrome, neurological and paralysed from waist down	Clawed hands unable to turn head to see
**A4**	4	0.5	0.5	Motor neuron disease	Rear visibility, kerbs, obstructions
**A5**	4	16	6	Spina Bifida	Spatial awareness
**A6**	4	0	12	Osteoarthritis	Pedestrians not seeing me, uneven paths/roads and slopes
**A7**	4	0	10	Ehlers-Danlos syndrome	Tiredness, poor proprioception, dislocation
**A8**	5	20	15	Myasthenia gravis	tiredness/weakness/not able to use on a bad day
**A9**	1	5	2	Cerebral palsy affecting all four limbs	jerkiness of my arms
**A10**	1	15	2	Cerebral ataxia	spatial awareness
**A11**	4	0	2	Poor balance/knees	concentration/not able to use on a bad day

**Figure 2.**
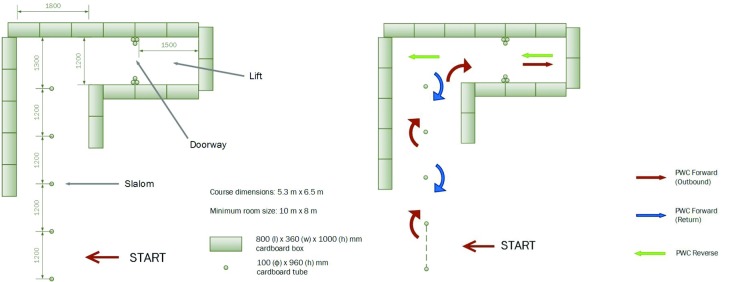
Participant driving evaluation course.

The second approach was to use data obtained from twelve participants (B group) who were invited to undertake evaluation of a smart PWC collision avoidance system (Dynamic Localised Force Field method ^14^) using a specially designed course to test manoeuvring in confined spaces in accordance with the ethical approval of the project. We also invited two student nurses to undertake the same course, who had never driven a PWC and had no disabilities, as a comparative control (labelled B5 and B6). All participants used the same PWC (Invacare Spectre XTR2 platform using a Dynamic Controls DX2 joystick control system) with the same driving profile and undertook the same path around the course, shown in Figure 2. They were asked to drive around the course as quickly as possible without colliding with any of the walls or posts as if they were on a competitive driving test. Their joystick control input to the PWC system, and the data from the collision avoidance ultrasound sensors measuring the range to the surrounding obstacles, was recorded by our monitoring hardware as they negotiated the driving course in chronologically labelled order of the participant participation. The anonymised B group participants’ backgrounds are given in Table 4. All participants were able to adjust their own upper body posture and had no additional means of support other than the standard PWC.

**Table 4.** Powered wheelchair user test course data recording, background.

Identifier	Driving ability	Years driving PWC	PWC driving training	Reason for PWC	Difficulties
**B1**	5	15	None	Cerebral Palsy	None at present
**B2**	5	3.5	Yes	Stroke	Left side paralysis
**B3**	5	11	Scooter	Duchenne muscular dystrophy	Muscular weakness
**B4**	5	9	None	Duchenne muscular dystrophy	Muscular weakness
**B5**	0	0	None	Control subject	None
**B6**	0	0	None	Control subject	None
**B7**	4	2	None	Brain injury	Attention, memory, anxiety, fatigue, equilibrium
**B8**	4	15	None	Multiple Sclerosis	Muscular weakness, spasticity
**B9**	4.5	16	Yes	Multiple Sclerosis	Muscular weakness, spasticity, equilibrium
**B10**	2.5	1	Yes, currently	Brain injury	Attention, planning, fatigue, equilibrium, visual, fine motor
**B11**	3.5	15	None	Cerebral Palsy	Left side spasticity
**B12**	2	2	Some	Not answered	Not answered
**B13**	4.5	4	None	Multiple Sclerosis	Not answered
**B14**	4.5	12	None	Tetraplegia	Fine motor control of fingers, left side weaker

The third approach was to obtain joystick movement data from two participants, B1 & B2, over a longer period of time (C group) without any modification to their control input. This was achieved by mounting on a standard Invacare Spectra PWC using a Dynamic Controls DX2 joystick control system we supplied, a joystick recording device connected to the manufacturer’s control system, and two buttons for the user to press, one for deliberate collision and one for accidental collision, an IMU, and a real time clock. The participants took turns to drive the PWC without any collision avoidance in their normal daily environment for at least three days. The inertial motion of the platform, joystick input, time-of-day, driving time, and the output from two manual digital buttons for identifying deliberate and accidental collisions were recorded on an SD card, without identifying what the user was doing and where they were. The identifier labels of B1 and B2 are respectively also C1 and C2 where the first letter simply relates to the different testing group and environment.

The participants in both group B and C were informed in writing, and verbally, what they would be volunteering to undertake, and how their data would be used for research and publication, for which they gave their written consent. All data has been anonymized without distortion or alteration to the scientific meaning.

### Feature development

Determining the user’s physical input range and rate of change of position to set-up the control system has been until now an iterative empirical process. In addition there also remains the issue of the user input ability changing over time and how that is monitored so that the system can be re-adjusted at some later date. A smart PWC system would need to analyse the user input characteristics and then adjust the control system mappings, in order to provide a progressively proportionally scaled robust and safe assistance. A further requirement of any smart assistive system ^10, 16^ is that the user is kept in control for as long as possible, rather than the smart system simply taking over control and confronting the user with an autonomous system. Therefore the first step in developing an adaptive assistive system is to identify the input profile of the user so that the joystick mapping can be better initially set-up and adjusted over time.

Returning to our initial feature set, given in Table 2, we hypothesise that we can identify the user input profile from their physical input quality, joystick position range, and rate of change of joystick position, and by feedback from obstacle proximity sensors we can also identify their visual spatial awareness. Thus essentially we can profile the user driving trajectory input and how it changes over time by monitoring the following features:

• Joystick Position
  ○ Biases/areas/quadrants
  ○ Range/magnitudes
• Joystick Velocity (actual user velocity of the joystick movement)
  ○ Sudden large magnitudes
  ○ Measure of smoothness
  ○ Biases/areas/quadrants
• Proximity to Obstacles
  ○ Biases
  ○ Magnitudes
• Time
  ○ Actual driving time day-to-day (long term trend)
  ○ Specific task
           ■ Overall
           ■ Ratio of moving to stopping
• Frequency of tremor/shake in joystick motion
  ○ Long term trend
  ○ Short term task/place specific

***Joystick input tremor and smoothness.*** Hand tremors can affect the joystick input quality, such that the user finds it challenging to operate a normal PWC. Therefore modern PWC control systems can be programmed to compensate for tremor. However the severity of the tremor may change over time such that sometimes the user may be fully capable of safely controlling the PWC and at other times could be potentially a danger to themselves and others around them. Research has identified the dominant tremor frequency is age related; particularly people with large amplitude tremors undergo a reduction in frequency as they age ^24^. Hand tremor frequency may also be dependent on the task; research suggests the displacement amplitude may decrease the tremor frequency by 3–4 Hz where the range is commonly between 4–11 Hz ^25^. One proposed solution was to develop Isometric Joysticks, which measure applied force rather than movement ^26^; however there was only a small improvement in performance.

A smart adaptive system would need to determine when the joystick signal input quality with adapted filtering is suitable to be acceptable as a proportional input, or whether treating the joystick as a switched device is a better option with the system providing appropriate assistance to control speed and rate of turn, acceleration and deceleration. However the first step in the process is to detect the presence of tremor, spasm or panic.

In order to detect tremors a Fast Fourier Transform will be used to determine if the joystick signal is sufficient for the purpose of monitoring the user for signs of tremor such that we can correct their joystick input:

$$\text{X}(\text{k})=\Sigma_{\text{j}=1}^{\text{N}}\text{x}(\text{j})\omega_{\text{N}}^{\text{(j}-1\text{)}(\text{k}-1)}\;(3)$$

Where:

                                                                                                                                    ω _N_ = e ^(−2πi)/N^                               (4)

Smoothness can be regarded as a measure of intended movement and jerk (ms ^-3^) has been commonly used as an empirical way to obtain some objective measurement of this feature. However several studies have reported mixed results with different jerk algorithms ^27^, such that dimensionless jerk and the log of the dimensionless jerk being the only valid measures of smoothness ^28^. However velocity (ms ^-1^) rather than jerk is thought to be a more appropriate measurement of smoothness ^29^ which in this case can be easily obtained from the standard joystick. The sample rate from the Dynamic Data Bus is set by the manufacturer at 50Hz. This rate is high enough to meet the requirement for smoothness measurement ^29^. Smoothness can be obtained by using the joystick velocity vector as follows:

$$\Lambda =\frac{\sum_{\text{i}=1}^{\text{n}}{\text{w}}_{\text{i}}\lambda_{\text{i}}}{\sum_{\text{i}=1}^{\text{n}}{\text{w}}_{\text{i}}}\;(5)$$

Where:

$$\lambda_{\text{i}}=\sqrt{\frac{{\left({\text{v}}_{2}-{\text{v}}_{1}\right)}^{2}*{\left(\omega_{2}-\omega_{1}\right)}^{2}}{\left({\text{t}}_{2}-{\text{t}}_{1}\right)}}\;(6)$$

And where (v _2_ − v _1_) is the distance the joystick has moved in the velocity plane and ( *ω*_2_ − *ω*_1_) is the distance moved in the turn plane, and t _2_ − t _1_ is the sample time period and the weight w _i_ is given by:

                                                                                                                                w _i_ = λ̃ _{1,2,.... *i*}_                                    (7)

In addition to tremor and smoothness there is the issue of user sudden movement or panic in response to a miscalculation or some involuntary muscular action when navigating around obstacles. The Peaks method (sudden direction change), which can be used in real-time, offers the potential for determining sudden jerks (panicky motion) or spasms as well as long term tremor monitoring.

**Figure 3.**
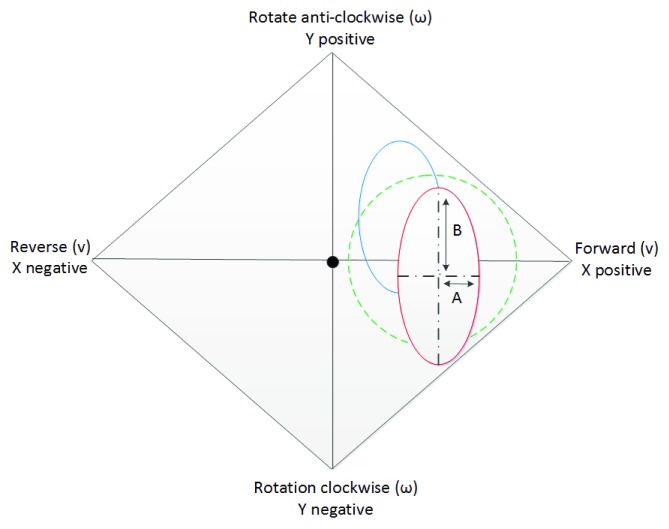
Joystick pattern and input ranges.

***Joystick positional bias.*** The user may need to have the joystick forward and reverse swapped because their ability to pull their hand towards them is better than to push the joystick away from them. They may also only be able to move the joystick part-way rather than the fully available range. This range, or throw, may also change over the day and over days. The general input pattern of the joystick can be said to lie within the diamond shape given in Figure 3, where the physical restriction of the device keeps the input within the kinematic boundary given by equations one and two. Additionally the software VRP, or equivalent, ensures the input continues to obey the boundaries when the throw shape is altered.

It is proposed that in order to identify measure and adjust the mapping the joystick input is represented by quadrants within which the shape of the user input profile is represented by shape parameters A and B, shown in Figure 3, such that they represent the semi major axis and semi minor axis of an ellipse where the joystick speed and turn (x and y) statistical position density distribution mid 50% inter-quartile range (IQR) represents the magnitude of A and B, and the centre point in the (x) axis is the median of the IQR, and the centre of the (y) axis is the first quartile.

In Figure 3 the user’s forward left quadrant might look like the blue ellipse and the forward right quadrant might be indicated by the red ellipse where an optimally mapped profile, for the task in hand, might look like the green ellipse where both quadrant ellipses are now overlapped. It should be noted that although the ellipse for the two forward quadrants crossover the actual position data is all in the respective quadrants, showing the full ellipse (overlap) is designed to permit a visual miss-alignment between how the user moves the joystick left/right, this is likely to be highly dependent upon how the user holds the joystick as well as the muscular flexibility (future research).

The position profile can be remapped to meet the needs of the user by either changing the pre-set profile velocity ranges (for specific tasks) or for a smart adaptive assistive system by setting the maximum ‘best day’ performance and then adjusting the mapping by taking the A and B parameters and using their spread to remap the input joystick values. This is done by taking the joystick velocity and turn commands off of the powered chair communication bus, remapping those values and returning them to the bus ^30^. The equation for remapping can be given by:

$$x_{out}=\frac{(x_{in}-in\_min)*range\_out}{\left(range\_in+out\_min\right)}\;(8)$$

Where the:

remapped value = x _out_

original value = x _in_

remapped output range (minimum to maximum) = range_out

original input range (minimum to maximum) = range_in

lowest or start value of the original input range = in_min

lowest or start value of the remapped output range = out_min

***Velocity vector bias.*** Whilst there may be a method to solve or filter the tremor/smoothness there may be also be a bias to the smoothness which can be because of muscular or motor neuron disease or due to visual neglect. Therefore it is proposed to determine the velocity vector in each quadrant by measuring the rate of change of the joystick position and to represent this as a range using the 50% IQR. This feature can be used to bias the Joystick input tremor and smoothness section for each quadrant where the velocity vector is broken down into components to adjust each axis in each quadrant. This would give the acceleration parameter range for the feed-forward control set-up and for the smart adaptive system to adjust as it changes over time.

***Proximity to obstacles.*** One crucial feature to consider for the adjustment of any assisted navigation system, must be the proximity to obstacles as the user manoeuvres around them. Whilst it is impossible to identify intent there is clearly a need for users to come into contact with obstacles, such as when transferring to a bed or chair, and when opening a manually operated door. Therefore the measurement of obstacle proximity must be one which identifies a bias in the pattern, such as driving very close to obstacles on the left side as opposed to the right side and more collisions in one particular sector around the user. This could indicate a visual or spatial awareness problem. This measure could be obtained by comparing the assisted system corrected user input with the actual input by taking a moving average of the differences in each sector.

$$F_{d}=1-\frac{1}{{\text{exp}}^{\left((R-p)/k\right)}}\;(9)$$

**Figure 4.**
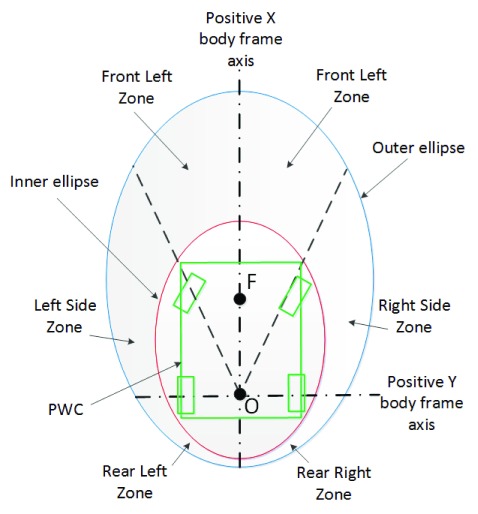
Collision avoidance zoning.

The Dynamic Localised Adjustable Force Field (DLAFF) ^14^ is one such collision avoidance method which can be dynamically adjusted according to user needs and abilities. The concept is based upon two travelling ellipses, as shown in Figure 4, which surround the PWC platform, the inner ellipse provides a zone within which the physical platform and user is located, the outer ellipse provides the limit of the damping force (F _d_), which is given by Equation 9, and which acts between them in a radial fashion about the mid-point of the rear PWC axle, marked ‘O’ in Figure 4, where R is the distance along the radial vector to the outer ellipse and p the distance along the radial vector to the inner ellipse, This value is then mapped, also using Equation 8, such that the range of y values (1 to 0.01) given by Equation 9 fits along the radial vector between p and R. This value of F _d_ then acts as a multiplier to damp the platform motion where the nearest forward right obstacle damps the left wheel motor input signal and the nearest forward left obstacle damps the right wheel motor input signal.

**Figure 5.**
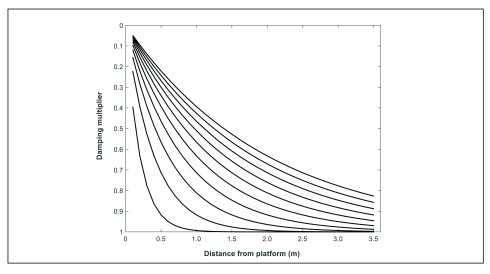
Adjustment the repulsive field of the collision avoidance by using the (k) value.

The size and shape of the two ellipses can be dynamically changed providing that one of the foci remains at the body origin ‘O’ and the other foci ‘F’ is constrained to the X body axis. The ellipse can also be extended outwards along the Y axis such that the repulsive zone each side can be extended or retracted. Areas of the ellipses can be sectioned into zones in a similar way to the joystick, such as shown in Figure 4; however in this case the zones should relate to the platform dynamic and kinematic such that the collision avoidance can be biased and adjusted over time. For example the user may have their left leg in plaster and in this case the inner ellipse foci ‘F’ would be moved outward such that the physical dimensions of the platform and user were kept within the inner ellipse. Another example might be that the user has poor vision to the left and has trouble negotiating obstacles to the left, in this case we might extend the outer ellipse on the left side and/or we might adjust the repulsion proportionality such as by changing the k value of Equation 9, as shown in Figure 5, to alter the collision avoidance behaviour to provide more or less safety distance between the platform and the obstacle. Altering the damping factor in this case will change the platform trajectory earlier in such a way that the obstacle is passed at a further distance than would otherwise have been the case, this is crucial in the case where the user may be travelling quicker than the system can respond.

***Duration of active PWC driving.*** The PWC user may be seated in their chair for long periods of time ^31^, effectively living in it. The time they spend out of bed, or other seating, and in their chair will be related to their day-to-day ability. Additionally the amount of time spent driving, rather than just sitting in the PWC may also indicate their current capability; however these may be long term health related features. For the purposes of adapting and adjusting any driving assistance time based features, the time driving to time stationary and the number of pauses when negotiating obstacles may have some relationship to levels of tiredness and reasoning and thus some direct relationship to the amount of assistance which is required.

### Feature evaluation and data processing

An adaptive system would require a metric of the user’s changing abilities over time, such that by using pattern recognition techniques these changing features can be identified. Essentially, a pattern of features is assigned to a particular event or symptom, and a classifier tries to match the current observation with saved patterns. Similar pattern recognition work to that required for a smart PWC adaptive system has been previously undertaken for various online classifiers to determine the suitability for developing real-time embedded systems. This research concluded that linear and quadratic discriminant analyses are highly suited and K-Nearest Neighbour-1 is possible if the training set does not become too large to the task ^32^ Naïve Bayes, Support Vector Machine, and Artificial Neural Networks proved were even more suitable ^33^. The research was undertaken on a dual core 1500 MHz 2GB RAM MICROSPACE EBX (MSEBX945) small computer format board with 1000 sample training set and 400 features. Another research project used a Weightless Neural Networks to classify simple geometric patterns in the microsecond time frame on an Atmel AT89x55 24.3MHz processor with 256 bytes of RAM ^34^. Therefore, in line with developing a real-time adaptive assistive system, we propose to evaluate the driving features by using the following classifiers:

- Linear Bayes Normal ^35^
- Fisher's linear discriminant ^36^
- Logistic linear ^37^
- Naive Bayes classifier ^38^
- Support vector machine ^39^
- Parzen classifier ^40^
- k-nearest neighbour ^41^

Features used for all of the fixed course monitoring of group B:

- FFT dominant frequency
- Smoothness
- Forward left ‘A’ from joystick position ellipse
- Forward left ‘B’ from joystick position ellipse
- Forward right ‘A’ from joystick position ellipse
- Forward right ‘B’ from joystick position ellipse
- Rear left ‘A’ from joystick position ellipse
- Rear left ‘B’ from joystick position ellipse
- Rear right ‘A’ from joystick position ellipse
- Rear right ‘B’ from joystick position ellipse
- Forward left velocity vector median
- Forward right velocity vector median
- Rear left velocity vector median
- Rear left velocity vector median
- Forward left collision bias
- Forward right collision bias
- Rear left collision bias
- Rear right collision bias
- Ratio of time in motion to time stationary
- Total course time

Features used for all of the three day monitoring of group C:

- FFT dominant frequency
- Smoothness
- Forward left ‘A’ from joystick position ellipse
- Forward left ‘B’ from joystick position ellipse
- Forward right ‘A’ from joystick position ellipse
- Forward right ‘B’ from joystick position ellipse
- Rear left ‘A’ from joystick position ellipse
- Rear left ‘B’ from joystick position ellipse
- Rear right ‘A’ from joystick position ellipse
- Rear right ‘B’ from joystick position ellipse
- Forward left velocity vector median
- Forward right velocity vector median
- Rear left velocity vector median
- Rear left velocity vector median
- Ratio of time in motion to time stationary

## Results

### Collision analysis

The ‘A’ group of volunteer participants (A1-A11) were asked to monitor their daily routine on a ‘good’ and ‘bad’ day. They were tasked with noting how many collisions occurred and what class of collision they were. Class (A) denotes accidental collisions, Class (D) refers to intentional and deliberate collisions such as attempting to use the PWC to open doors, and class (C) relates to directional changes where the user has needed to reverse and re-approach due to initial misjudgement of the correct alignment to a doorway for example. Participants A8 and A11 were unable to drive the PWC on a bad day.

The results of the good day collisions, shown in Table 5, clearly show that using the PWC to push open doors is quite common, however the number of accidental collisions with the door frame was high compared to deliberate collisions and misalignments. In comparison with the same two types of obstacle on a bad day, it can be seen that the number of deliberate collisions did not change significantly, yet the accidental collision and misalignment incidents rose to be of similar occurrence, with door frame misalignments doubling ( Table 6). This pattern was similarly followed with the other types of obstacles, with a general increase in the number of accidental collisions, in particular with that of the misalignment class.

**Table 5.** Collisions on a good day.

Obstacle	class	Identifier											Totals
		A1	A2	A3	A4	A5	A6	A7	A8	A9	A10	A11	
**Door**	*A*	0	0	2	0	3	0	0	1	0	0	1	***7***
	*D*	0	2	6	2	5	0	4	0	0	0	0	***19***
	*C*	0	0	2	0	2	4	1	0	0	0	N/A	***9***
**Doorway** **frame**	*A*	0	1	2	1	10	0	0	2	4	10	2	***32***
	*D*	0	0	1	0	0	0	0	0	0	0	0	***1***
	*C*	0	0	3	1	2	2	0	0	0	0	0	***8***
**Wall**	*A*	0	0	0	1	4	0	0	0	0	5	1	***11***
	*D*	0	0	0	0	2	0	0	0	0	0	0	***2***
	*C*	0	0	1	1	0	0	1	0	0	0	0	***3***
**Furniture**	*A*	0	0	0	1	3	0	1	1	4	10	0	***20***
	*D*	0	0	0	0	0	0	0	0	0	0	0	***0***
	*C*	0	0	1	0	1	2	2	0	0	0	0	***6***
**People**	*A*	0	0	0	0	1	0	0	0	2	3	0	***6***
	*D*	0	0	0	0	0	0	0	0	0	0	0	***0***
	*C*	0	0	2	0	2	5	0	0	0	0	0	***9***
**Road**	*A*	0	0	1	1	3	0	0	2	1	5	1	***14***
	*D*	0	0	1	0	4	0	3	0	0	0	0	***8***
	*C*	0	0	1	0	1	0	0	0	0	0	0	***2***
**Totals**		***0***	***3***	***23***	***8***	***43***	***13***	***12***	***6***	***11***	***33***	***5***	***157***
**Hours in PWC**		16	18	8.5	5	16	10	12	12	5	N/A	2	***104.5***

**Table 6.** Collisions on a bad day.

Obstacle	Class	Identifier											Totals
		A1	A2	A3	A4	A5	A6	A7	A8	A9	A10	A11	
**Door**	*A*	1	0	5	0	7	0	2	N/A	4	0	N/A	***19***
	*D*	1	2	4	2	3	0	10	N/A	0	0	N/A	***22***
	*C*	1	0	5	2	4	2	3	N/A	0	0	N/A	***17***
**Doorway** **frame**	*A*	1	1	6	5	6	0	3	N/A	8	5	N/A	***35***
	*D*	1	0	1	0	1	0	0	N/A	0	0	N/A	***3***
	*C*	1	0	6	2	2	0	4	N/A	0	0	N/A	***15***
**Wall**	*A*	1	0	2	1	10	0	2	N/A	0	1	N/A	***17***
	*D*	0	0	1	0	2	0	0	N/A	0	0	N/A	***3***
	*C*	0	0	3	2	1	1	6	N/A	0	0	N/A	***13***
**Furniture**	*A*	1	0	2	4	11	1	2	N/A	8	5	N/A	***34***
	*D*	0	0	1	0	2	0	0	N/A	0	0	N/A	***3***
	*C*	0	0	4	2	1	2	5	N/A	0	0	N/A	***14***
**People**	*A*	0	0	5	1	2	1	1	N/A	4	0	N/A	***14***
	*D*	0	0	1	0	0	0	0	N/A	0	0	N/A	***1***
	*C*	0	0	1	0	2	9	6	N/A	0	0	N/A	***18***
**Road**	*A*	0	0	3	3	4	3	1	N/A	2	1	N/A	***17***
	*D*	0	0	1	0	2	0	4	N/A	0	0	N/A	***7***
	*C*	0	0	1	0	1	6	2	N/A	0	0	N/A	***10***
**Totals**		***8***	***3***	***52***	***24***	***61***	***25***	***51***	***N/A***	***26***	***12***	***N/A***	***262***
**Hours in PWC**		12	18	4.5	7	16	15	16	N/A	2	N/A	N/A	***90.5***

In addition to gathering data via the volunteer questionnaires, two experienced PWC users were monitored over an extended period of time, one for five days and the other for four days. In this case they used a standard powered chair, which included an electronic data collection system. This system enabled the users to record collisions by pressing a button. The data indicated that on average for each hour in the PWC, C1 had 3.9 and C2 had 3.3 accidental collisions ( Table 7). When this was averaged against the actual driving time the rate increased to: C1 had 12.3 and C2 had 7.2 collisions per hour of actual driving. The average collisions recorded by the 11 questionnaire participants showed an average accident rate of around one per hour.

**Table 7.** Long term collision data.

		Day			
		1	2	3	
Id	Class				Totals
**C1**	***Number of deliberate collisions***	3	1	1	5
	***Number of accidental collisions***	3	17	23	43
	***Hours in PWC***	5.45	3.13	3.03	11.61
	***Hours driving***	1.04	0.83	1.63	3.5
**C2**	***Number of deliberate collisions***	4	0	1	5
	***Number of accidental collisions***	14	8	32	54
	***Hours in PWC***	5.2	0.97	10.35	16.52
	***Hours driving***	2.98	0.73	3.79	7.5

The response from the participant questionnaire is given in Table 8 and this indicated that there was a marked shift of abilities between a ‘good day’ and a ‘bad day’. The range of abilities stretched from being reasonably able to function to needing full support from carers.

**Table 8.** Range of symptoms between good and bad days.

ID	A			B			C			D			E			F			G			H		
	g	b	d	g	b	d	g	b	d	g	b	d	g	b	d	g	b	d	g	b	d	g	b	d
**A1**	1	1	0	1	1	0	1	1	0	1	1	0	2	4	2	2	4	2	1	3	2	1	1	0
**A2**	1	1	0	1	1	0	1	1	0	1	1	0	1	2	1	1	2	1	1	3	2	1	1	0
**A3**	2	4	2	2	3	1	2	3	1	2	3	1	4	5	1	4	5	1	4	5	1	2	4	2
**A4**	1	1	0	1	1	0	1	1	0	1	1	0	2	2	0	2	5	3	2	5	3	1	1	0
**A5**	1	1	0	1	2	1	1	3	2	1	1	0	1	3	2	1	3	2	2	4	2	2	4	2
**A6**	1	3	2	1	3	2	1	3	2	1	3	2	1	4	3	1	3	2	1	4	3	1	3	2
**A7**	1	3	2	1	3	2	1	3	2	1	3	2	2	3	1	2	5	3	1	4	3	1	3	2
**A8**	1	1	0	1	2	1	1	2	1	1	2	1	1	3	2	3	5	2	3	5	2	2	5	3
**A9**	3	5	2	1	3	2	2	4	2	1	3	2	3	5	2	3	5	2	3	5	2	2	4	2
**A10**	1	3	2	3	5	2	2	5	3	3	5	2	1	2	1	3	5	2	3	5	2	3	5	2
**A11**	3	4	1	2	3	1	1	2	1	1	2	1	2	3	1	2	3	1	3	4	1	1	2	1

Key: A1:A11 = Participant identification g = Good day b = Bad day d = Difference between a good day and a bad day A to H = Symptom identifier given in Table. 4. 1 = not *suffering* with this (within normal range) 2 = causes occasional problems 3 = problematic effecting day-to-day tasks 4 = severely affecting personal performance 5 = unable to function without assistance

**Key for Table 8.** 

Class	Symptom
**A**	Muscular tremors and/or spasms
**B**	Attention and/or concentration difficulty
**C**	Panic and/or agitation (nervousness)
**D**	Reasoning and/or confusion
**E**	Muscular stiffness
**F**	Muscular weakness
**G**	General fatigue/tiredness
**H**	Observational and/or visual bias

### Joystick tremor and smoothness analysis

An experiment was undertaken, with the view of establishing whether the data rate from the standard commercially available system was sufficient to measure both the tremor and smoothness features. Commonly the Normalised Jerk score is employed ^27^ to measure smoothness; however this is the third derivative of joystick position whereas velocity is the first order. This would mean that using jerk to score smoothness, rather than velocity, would require a higher sample rate than is available.

**Figure 6.**
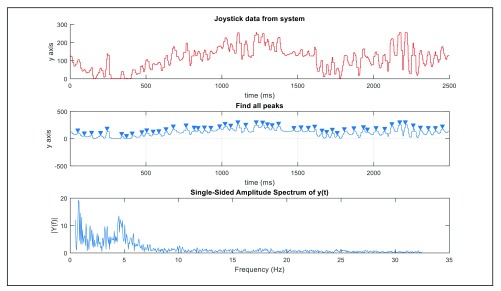
Example plot of the digital system joystick data frequency feature analysis.

An artificial tremor was analysed to obtain the frequency by using the two methods as previously described. The first involved using a Fast Fourier Transform and the second counting peaks in the signal as it changed sign over time, an example is shown in Figure 6. As expected the 50Hz sample rate from the commercial system is suitable for measuring the typical tremor rate range; however the notched joystick plot, and experimentation, indicated that it would not be suitable for third order differentiation to obtain jerk.

In order to determine if velocity is a good measure of joystick smoothness we needed to compare this with the traditional Normalised Jerk Score; therefore a three axis accelerometer was mounted into the joystick handle and additionally, an analogue to digital converter was connected directly to the Hall Effect sensor coils and samples from both sensors were acquired at a data rate of ≈250Hz thus directly measuring acceleration and velocity. Joystick position data was also collected at 50Hz from the PWC data bus (Dynamic Controls DX2) using their proprietary interface ^30^.

A series of 16 artificial tremors at different frequencies were physically generated and the data from the accelerometer, analogue joystick, and digital joystick from the system bus recorded. The data was then first analysed to determine whether velocity obtained from differentiating the joystick position data was a reasonable feature for determination of smoothness.

The joystick movement data from the accelerometer and from the system data bus were compared to determine the suitability for extracting the frequency from the standard PWC data bus. In addition, the velocity vector was derived from both the joystick data and the much higher sample rate directly from the joystick coils; this was compared to the traditional method of obtaining the weighted average jerk from accelerometer data. Both sets of data are given in in Table 9.

**Table 9.** Joystick multimodal frequency analysis.

	FFT accelerometer y axis (Hz)	Magnitude dominant y axis frequency	FFT Joystick digital y axis (Hz)	Frequency digital peak count (Hz)	Weighted average (x, y) jerk vector	Analogue velocity (x, y) vector smoothness	Digital velocity (x, y) vector smoothness
**1**	3.523	0.247	3.197	3.619	0.296	0.081	0.159
**2**	6.016	3.048	5.908	6.471	2.619	0.121	0.715
**3**	4.715	0.488	4.661	4.920	0.586	0.082	0.219
**4**	3.957	5.981	3.957	4.861	4.440	0.205	1.418
**5**	3.957	4.841	4.282	4.486	2.129	0.136	0.865
**6**	3.360	9.117	3.36	3.842	3.724	0.217	1.613
**7**	4.390	3.619	4.39	5.050	2.118	0.130	0.800
**8**	8.618	0.599	7.859	6.767	1.130	0.077	0.198
**9**	4.471	1.738	4.444	4.971	1.333	0.093	0.447
**10**	6.178	3.802	5.85	5.366	5.767	0.204	1.341
**11**	4.498	3.469	4.498	5.218	4.166	0.177	0.996
**12**	4.336	0.191	4.336	3.558	0.361	0.082	0.157
**13**	4.878	0.242	4.878	2.521	0.312	0.080	0.150
**14**	4.444	0.810	4.444	4.817	0.445	0.083	0.184
**15**	3.144	1.438	3.144	4.999	0.715	0.082	0.262
**16**	6.585	0.316	6.097	3.650	4.042	0.245	1.951

The accelerometer and joystick bus values were compared using ANOVA to determine whether the joystick bus data rate was sufficient to determine the tremor frequency by using either the FFT or digital peak count methods. The FFT method digital joystick compared to accelerometer gave F (15, 15) = 108, p = 0 where F critical = 2.4. The digital joystick peak method returned F (15, 15) = 2.79, p = 0.028 where F critical = 2.4. Therefore the FFT method is very good and the peaks method is a fair method of measuring tremor when taking joystick data from the system bus compared to using an accelerometer.

**Figure 7.**
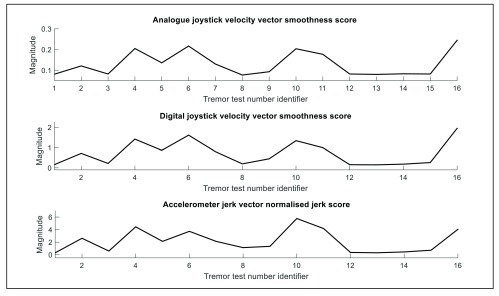
Comparison of the smoothness score with the normalised jerk score using an accelerometer, analogue measurement of the velocity vector direct from the joystick coils and the digital data from the PWC bus.

There appeared to be little correlation between the different methods of evaluating velocity vector smoothness when the data was initially reviewed, however this was due to the sampling rate and hence scaling. All three methods are given for comparison in Figure 7. Therefore if we multiply the velocity vector smoothness obtained from the joystick digital data by a factor of two, to try to adjust the scale difference, we can compare the digital joystick directly with the weighted average jerk to determine if there is a significance. This gave a digital smoothness score to accelerometer normalised jerk score correlation of F (15, 15) = 10.7, p = 0 and F critical = 2.4 which indicated that there was a significant correlation between the two methods. Therefore the joystick data obtained from the PWC system can be used with the velocity smoothness algorithm to determine the user smoothness of input without the need to mount additional sensors.

**Figure 8.**
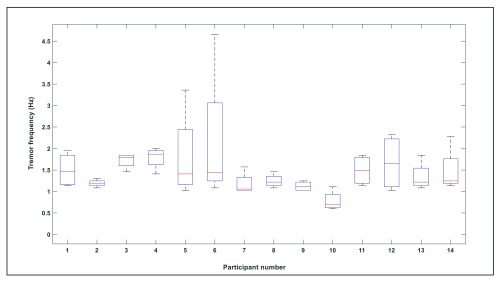
Tremor frequency comparisons for all fixed course participants.

**Figure 9.**
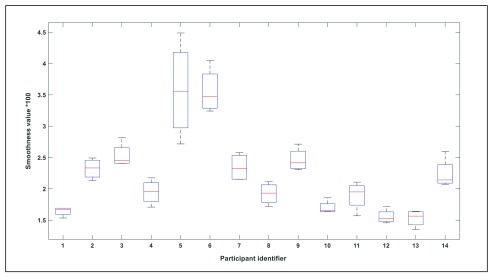
Smoothness comparisons for all fixed course participants.

The tremor frequency and range of the fixed course participants, depicted in Figure 8, showed that most of the participants did not have a significant tremor. Only two of the participants appeared to have a significant tremor; however both were young student nurses who had never driven, or attendant operated, a PWC who were asked, without prior warning, to take part as novice, non-disabled non-users for the purpose of comparison. They were both very nervous and anxious about undertaking the test course which we believed was the reason for the tremor and the smoothness results. The other participants had little variation in the range of smoothness ( Figure 9), although their individual ranges appeared to indicate that this might be an identifying feature for each individual.

**Figure 10.**
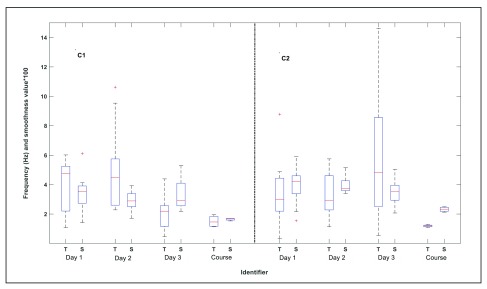
Tremor frequency (T) and smoothness (S) features variability day-to-day compared to specific task for participants C1 and C2.

The results in Figure 10 showed the three day range of tremor and smoothness for participant C1 and C2, who were also, B1 and B2 respectively. The participants undertaking the test course were instructed to complete the course as quickly as possible as if on a driving test. When the day to day user and their driving test course tremor and smoothness were compared it was clear that there was a much narrower range for the test course. Additionally, C2 reported feeling unwell and to have had difficulties getting around the outside rear of their house on day three.

There was a large range of tremor variation on the last day for C2, however the smoothness range remained similar to the other days unlike the two non PWC users (B5 and B6) on the test course who showed large variation in both smoothness and tremor when stressed. There remains a question as to whether the difficulty in operating the PWC for C2 was because of an increased tremor due to illness, or that the action of attempting to manoeuvre the platform gave rise to a tremor-like motion of the joystick or that the increased tremor and difficulty reported in manoeuvring the chair was a result of feeling unwell. We believe the latter is the case, otherwise smoothness would also have been affected.

### Joystick position analysis

The PWC user is likely to have different joystick usage profiles for different tasks. For example driving indoors in a highly cluttered environment will require more left to right movements to avoid objects compared with driving outside in open spaces which will not require so much correction. The user is provided with a range of programmed profiles to suite each environment. For example acceleration, deceleration, rate of turn and velocity will have relatively low values for indoor use and high values when in open spaces or outdoors. Ideally, an adaptive assistive system could use the user’s driving characteristics to recognise the environment in which the user is driving, and then automatically select the most appropriate profile for the user and micro-adjust as the user’s needs change over the day.

The results in the tables and figures abbreviate forward right ‘a’ component of the ellipse as ‘fra’ and rear right ‘b’ as rrb or ‘ra/la’ and ’rb/lb’ as right/left ‘a’ and right/left ‘b’, where not otherwise labelled.

**Figure 11.**
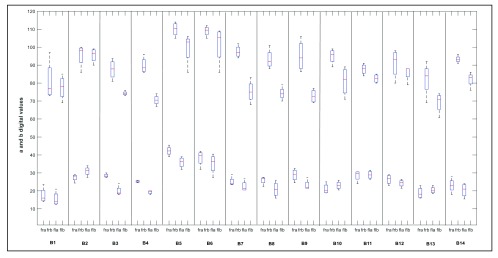
Joystick forward quadrants position pattern ellipse bias parameters for all fixed course participants.

The driving test course results for the different features are given in Table 10a and Table 10b, Figure 11 shows the forward quadrants only as the task was mainly forwards where the reverse element was specifically tasked into a left hand corner and therefore not enough data was present for proper analysis, although the results are still given in Table 10. This test course could be described as a highly cluttered indoor environment, therefore it would be expected that more time would be spent turning than driving in a pure forward or reverse direction due to the lack of free space. The data for the ‘C’ group for the three days was analysed in two ways, firstly the range of change over the day was broken down into segments of driving greater than 30 seconds over each of the entire day’s driving, given in Table 11 and Table 13, and secondly was to use all of the data combined for each entire day, Table 12 and Table 14.

**Table 10A.** Feature data from driving test course (median values).

Id	Run	FFT (Hz)	‘S’ *10 ^2^	Position bias ellipse								Velocity vector bias (mm/s)				Obstacle bias				Driving time	
				forward right/left				rear right/left				forward		rear		forward		rear			
				ra	rb	la	lb	ra	rb	la	lb	right	left	right	left	right	left	right	left	%	Total (s)
**B1**	1	1.73	1.69	17	74	13	76	10	44	10	13	8	11	13	18	27	32	34	41	80	111
	2	1.19	1.69	14	73	13	69	42	116	5	8	8	10	11	8	25	25	35	32	69	100
	3	1.14	1.65	24	97	21	85	23	76	17	119	8	8	8	15	34	28	45	32	99	146
	4	1.95	1.53	15	80	16	80	17	55	9	26	8	8	8	7	33	30	37	35	100	122
**B2**	1	1.19	2.24	29	100	31	95	25	72	10	22	11	13	8	10	33	31	34	31	99	145
	2	1.3	2.13	24	86	28	90	12	48	9	36	13	10	12	8	32	33	36	41	97	146
	3	1.08	2.49	29	97	34	99	5	25	21	55	12	13	11	20	33	29	34	44	98	143
	4	1.19	2.42	28	99	32	98	16	59	44	112	12	13	8	8	38	32	35	37	98	152
**B3**	1	1.84	2.41	29	90	18	73	17	31	8	25	11	11	10	18	37	33	38	35	100	119
	2	1.84	2.41	30	94	19	74	4	19	1	7	8	13	7	10	37	36	39	35	99	118
	3	1.46	2.5	28	81	19	76	9	35	4	16	8	15	9	14	40	33	36	42	96	110
	4	1.73	2.82	28	86	24	74	13	40	10	27	11	13	12	18	39	35	35	35	97	120
**B4**	1	1.41	2.18	25	96	20	71	21	63	34	84	11	13	10	37	30	36	38	32	98	116
	2	1.9	2.02	26	87	20	74	13	45	6	20	8	8	10	13	35	39	37	40	100	112
	3	2.01	1.71	26	90	18	67	15	55	1	8	8	8	8	12	31	39	38	46	99	110
	4	1.84	1.89	25	86	19	70	17	57	3	17	10	10	7	7	34	36	38	41	100	111
**B5**	1	1.03	4.49	42	105	32	86	55	127	42	120	21	14	7	31	35	41	37	31	79	125
	2	3.36	3.87	43	112	35	103	55	127	8	24	15	16	15	7	35	41	35	37	99	154
	3	1.3	3.23	46	114	39	103	21	50	54	126	12	15	5	12	35	37	40	27	100	130
	4	1.52	2.72	39	109	38	106	9	35	0	0	8	10	5	0	39	34	36	0	100	127
**B6**	1	4.66	4.05	42	110	35	103	43	127	22	44	13	10	12	18	38	42	25	34	66	115
	2	1.46	3.62	39	109	41	109	35	117	8	24	13	10	7	13	35	30	43	35	78	111
	3	1.08	3.24	41	112	38	108	14	41	21	48	11	8	8	26	33	31	36	36	91	122
	4	1.41	3.33	32	105	28	86	9	26	21	46	11	11	8	15	36	31	38	45	90	115
**B7**	1	1.03	2.16	24	96	22	68	6	28	26	10	12	12	6	17	31	31	41	23	100	137
	2	1.03	2.15	29	102	21	74	14	39	13	26	14	15	9	18	30	32	40	38	100	149
	3	1.57	2.58	24	98	27	83	22	50	17	61	15	15	9	33	31	33	38	26	100	159
	4	1.08	2.49	24	94	21	76	16	44	12	37	18	15	9	19	33	38	39	39	99	153

**Table 10B.** Feature data from driving test course (median values).

Id	Run	FFT (Hz)	‘S’ *10 ^2^	Position bias ellipse								Velocity vector bias (mm/s)				Obstacle bias				Driving time	
				forward right/left				rear right/left				forward		rear		forward		rear			
				ra	rb	la	lb	ra	rb	la	lb	right	left	right	left	right	left	right	left	%	Total (s)
**B8**	1	1.19	1.85	23	91	16	74	19	48	3	10	10	10	6	6	35	35	33	45	89	169
	2	1.25	2.02	27	101	22	70	8	18	26	6	10	10	6	39	40	33	38	25	95	150
	3	1.08	1.72	28	88	19	79	45	116	6	6	10	10	6	6	34	34	36	39	99	130
	4	1.46	2.11	27	93	26	74	40	94	22	10	10	12	6	10	38	32	38	36	97	142
**B9**	1	1.25	2.3	25	86	22	70	28	80	17	81	15	14	10	17	34	33	41	33	95	137
	2	1.03	2.34	28	106	22	69	20	62	0	4	14	11	9	4	30	35	43	38	96	136
	3	1.03	2.49	33	98	28	77	17	53	46	86	13	13	10	15	34	38	40	31	96	128
	4	1.19	2.71	31	90	22	75	28	80	24	25	15	15	11	12	35	36	41	34	94	137
**B10**	1	0.76	1.86	19	99	22	71	12	38	39	125	10	10	4	12	34	31	35	22	99	223
	2	1.11	1.64	25	89	24	86	41	100	11	42	10	7	9	12	29	30	30	36	100	190
	3	0.6	1.64	19	95	21	78	48	109	13	20	10	6	9	6	35	29	41	36	100	207
	4	0.65	1.67	22	97	26	89	25	69	28	33	9	10	6	6	29	28	33	30	100	198
**B11**	1	1.73	1.57	24	84	28	85	15	53	35	60	10	10	11	10	31	25	38	35	99	136
	2	1.84	1.91	31	87	32	84	14	44	39	91	12	10	10	13	29	32	37	49	96	140
	3	1.25	1.99	31	91	30	81	14	47	42	100	12	12	10	26	28	31	38	19	99	145
	4	1.14	2.11	29	89	27	80	23	66	33	8	13	12	9	27	29	29	38	40	98	134
**B12**	1	2.33	1.72	26	96	24	79	28	28	23	32	10	10	6	19	27	28	36	22	94	178
	2	1.03	1.55	28	98	25	88	19	18	3	127	10	9	10	4	28	30	38	29	99	158
	3	1.19	1.46	29	90	22	88	12	34	35	117	9	10	6	9	30	29	37	21	98	144
	4	2.11	1.51	23	80	26	88	21	40	16	124	10	10	9	9	25	28	37	18	99	133
**B13**	1	1.25	1.63	17	84	21	72	20	28	26	37	10	10	6	10	28	28	37	46	95	146
	2	1.19	1.64	20	84	20	70	20	44	46	111	10	10	10	10	28	30	34	12	91	145
	3	1.08	1.50	16	69	23	61	14	53	31	22	7	6	10	13	26	29	39	23	92	151
	4	1.84	1.35	23	92	19	74	10	29	7	1	10	6	4	29	27	30	37	10	94	143
**B14**	1	2.28	2.60	18	93	24	86	20	43	37	112	15	19	12	9	42	29	37	31	88	147
	2	1.25	2.11	23	91	23	83	14	46	40	122	14	12	10	10	34	32	39	17	99	131
	3	1.25	2.18	23	93	19	83	16	38	34	100	14	12	10	12	33	33	42	24	100	134
	4	1.14	2.07	28	96	16	76	18	59	13	13	12	14	9	15	35	33	48	40	100	137

**Table 11.** The range of variance of features for C1 for each day showing the minimum, median, and maximum values when the motion of the platform was at least 30 seconds in duration.

C1		Day								
		1			2			3		
**Feature**		min	med	max	min	med	max	min	med	max
**FFT Joystick digital ‘y’ axis (Hz)**		1.1	4.8	6.0	2.3	4.5	10.6	0.5	2.2	4.4
**Velocity vector (x, y) ×10 ^2^** **smoothness ‘S’**		1.4	3.5	6.1	1.7	2.9	3.9	2.2	2.9	5.3
**Input bias ellipse**	F/right a	6.5	19	44	6	13	24	12	18	24
	F/right b	13	20	104	18	34	60	15	29	71
	F/left b	5	18	35	6	10	21	8	16	32
	F/left b	14	21	97	21	36	70	20	26	102
	R/right a	3	16	49	6	20	40	16	25	36
	R/right b	18	43	127	6	56	96	22	31	102
	R/left a	8	13	35	2	17	32	10	18	47
	R/left b	16	30	76	28	57	127	18	72	90
**Velocity vector bias** **(mm/s)**	F/right	10	24	45	10	16	24	12	16	40
	F/left	13	25	33	11	19	29	10	15	42
	R/right	6	28	89	10	26	73	15	33	51
	R/left	9	21	160	11	21	46	14	23	36
**Driving time**	ratio	0.1	0.5	1.6	0.4	0.7	1.6	0.1	0.4	1.0
	total	39	95	360	30	75	161	56	121	1424

**Table 12.** Average daily feature values for C1 calculated from complete dataset of all motions.

C1			Day		
feature			1	2	3
**FFT Joystick digital y axis (Hz)**			0	0	0
**Velocity vector (x, y) ×10 ^2^ smoothness ‘S’**			3.5	2.9	4.4
**Position bias ellipse**	**forward**	right a	34	16	36
		right b	20	35	29
		left a	32	15	35
		left b	20	38	27
	**rear**	right a	26	22	28
		right b	44	59	62
		left a	23	22	25
		left b	37	59	69
**Velocity vector bias (mm/s)**	**Forward**	right	24	16	30
		left	25	18	33
	**rear**	right	14	20	28
		left	18	20	21
**Driving time**		ratio	0.7	1.1	0.4
		total	4507	3822	6722

**Table 13.** The range of variance of features for C2 for each day showing the minimum, median, and maximum values when the motion of the platform was at least 30 seconds in duration.

C2		Day								
		1			2			3		
**Feature**		min	mid	max	min	mid	max	min	mid	max
**FFT Joystick digital ‘y’ axis (Hz)**		0.3	3.0	8.8	1.1	2.9	5.7	0.5	4.8	14.6
**Velocity vector (x, y) ×10 ^2^ smoothness ‘S’**		1.5	4.2	5.9	3.4	3.7	5.1	1.3	3.5	5.1
**Input bias ellipse**	F/right a	7.5	14	50	0	22	50	0	13	39
	F/right b	5	88	127	15	62	127	7	88	127
	F/left b	5	16	56	11	15	29	7	15	47
	F/left b	18	95	127	8	102	127	7	98	127
	R/right a	0	29	46	4	22	41	4	30	49
	R/right b	13	88	124	10	50	127	8	76	127
	R/left a	1	25	36	3	23	29	1	26	44
	R/left b	8	95	116	3	108	125	7	91	127
**Velocity vector bias (mm/s)**	F/right	8	23	45	7	22	34	5	19	35
	F/left	8	25	78	8	21	46	5	23	89
	R/right	6	20	153	8	16	23	5	18	36
	R/left	9	22	41	7	16	21	6	19	88
**Driving time**	ratio	0.2	0.6	1.5	0.3	0.8	1.8	0.1	0.6	1.5
	total	35	91	1192	36	85	402	32	69	812

**Table 14.** Average daily feature values for C2 calculated from complete dataset of all motions.

C2			Day		
feature			1	2	3
**FFT Joystick digital y axis (Hz)**			0	0	0
**Velocity vector (x, y) ×10 ^2^ smoothness ‘S’**			3.9	4.0	3.6
**Position bias ellipse**	forward	right a	43	30	21
		right b	67	86	88
		left a	46	24	21
		left b	67	111	93
	rear	right a	42	45	38
		right b	83	81	74
		left a	28	33	32
		left b	93	112	91
**Velocity vector bias (mm/s)**	Forward	right	18	21	19
		left	21	22	22
	rear	right	19	15	16
		left	22	17	19
**Driving time**		ratio	0.7	0.6	0.6
		total	11261	2441	24364

**Figure 12.**
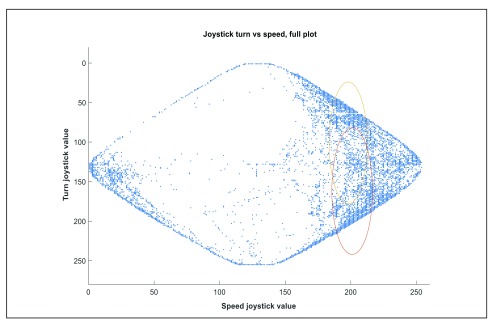
Joystick positional pattern for B1/C1 during test course.

**Figure 13.**
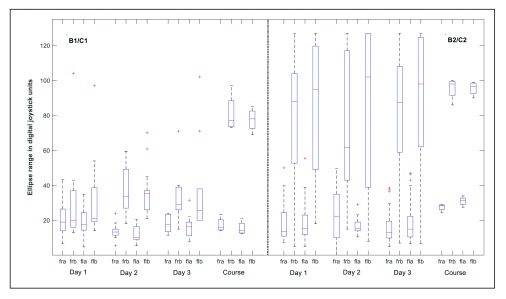
Joystick forward quadrant position pattern ellipse bias parameter over time compared to specific task for B1/C1 and B2/C2.

**Figure 14.**
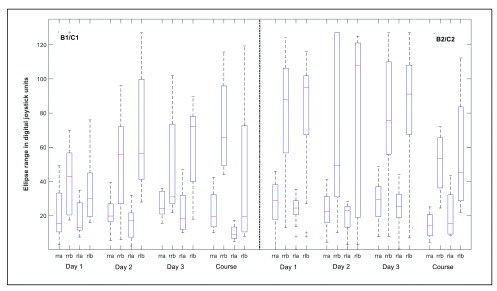
Joystick rear quadrant position pattern ellipse bias parameter over time compared to specific task for B1/C1 and B2/C2.

**Figure 15.**
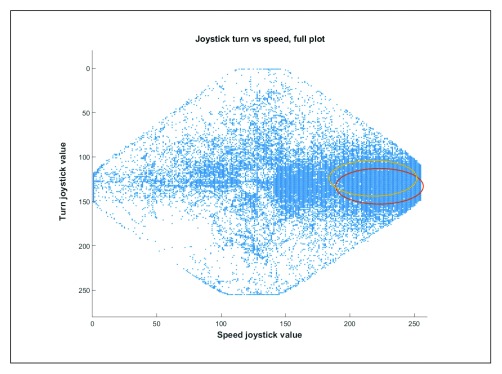
Positional bias plot showing forward left and right ellipses of participant C1 day 2.

The outcome indicated that the joystick turn position range was clearly not suited to the manoeuvring needs during the test course, the ellipse bias parameter ‘b’ being much larger and towards the limit of the left right throw range shown in Figure 11, for all of participants. This mapping can be clearly seen from the joystick ellipse profile of B1/C1 shown in Figure 12. However when we look at the extended three day experiment it can be seen from the data shown in Figure 13– Figure 14 that the settings given to the user were more suited to them for their environment, with the ellipse parameters plotted and shown in Figure 15. It is also clear when we compared the range of variance within the day and over the days for group C with their performance during the B group test course that joystick position was task specific, furthermore there appeared to be a range of operation specific to each individual user, as seen by the tightly clustered ellipse parameters in Figure 11, this indicated that this metric is a potential identifying feature as well as a means of adjusting the PWC initial profile mappings and to re-map them as their abilities change over the long-term.

### Joystick velocity vector bias analysis

**Figure 16.**
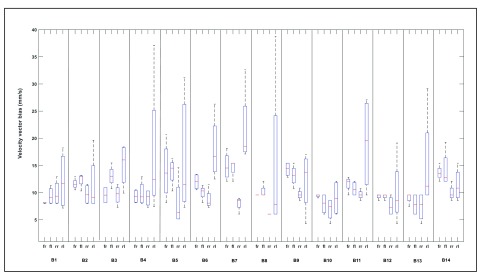
Velocity vector quadrant bias for all fixed course participants.

In addition to the PWC user joystick positional pattern and biases, due to physical and/or cognitive impairment, there is the issue of the rate of change of position. Where smoothness and tremor give an overall quality to the motion, there still remains the issue of which specific direction the motion needs more or less damping. For example a user may find pulling the joystick towards them is much easier than pushing away from them which results in different rate of change between driving forward and reverse which may change for the user over time. Whilst this research has simply depicted the velocity in quadrant vector form (forward-left (fl), forward-right, rear-left (rl), rear-right (rr)) in the box-plot format shown in Figure 16, it is expected in future that the vector will be split into turn and speed component form for adjustment of damping.

**Figure 17.**
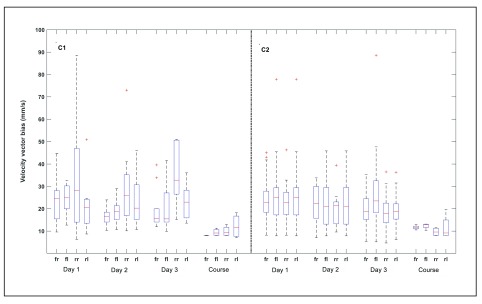
Three day velocity vector quadrant bias variability over time compared to specific task.

There is a certainty that in general reversing will not be as smooth as driving forward; although participant B2/C2 appeared to be equally smooth driving forward or backwards there was a slight reduction in the relative range of smoothness in the rear right compared to the rear left which may have been due to the user’s restricted movement on the left side whilst the participant B1/C1 appeared to have difficulty in the rear right quadrant according the data ( Figure 17).

### Obstacle proximity bias analysis

**Figure 18.**
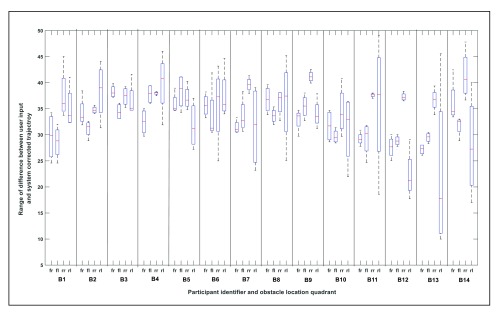
Obstacle avoidance quadrant relative bias for all fixed course participants.

An additional four laps of the driving course were undertaken with the collision avoidance system on so that any bias in the proximity to obstacles that a driver has as they pass that obstacle could be determined which may indicate some visual or perception difficulty; therefore it would be imperative to include this as a metric in any driving performance assessment and a definitive component for the adjustment of any navigation assistive system. The quadrant relative bias is shown in Figure 18, where the magnitude in the y axis denotes the amount of difference between the user joystick input and the system determined corrected joystick input calculated to keep the platform a safe distance from the obstacle, where the ‘k’ value was fixed permanently at the same value for all.

Participant B1 had a bias in the rear right quadrant which appeared to correlate with the velocity bias the user also had in that quadrant when they participated in the group ‘C’ trial. This relevance has more significance when we compare B1 with all of the other participants who appeared to have a greater level of system intervention bias towards the rear left side as would be expected when manoeuvring into a left hand corner. There was a significant range of intervention across all of the participants with some having had very little difference between system generated trajectories and their own and yet others had a large range of difference.

### Duration of active PWC driving analysis

**Figure 19.**
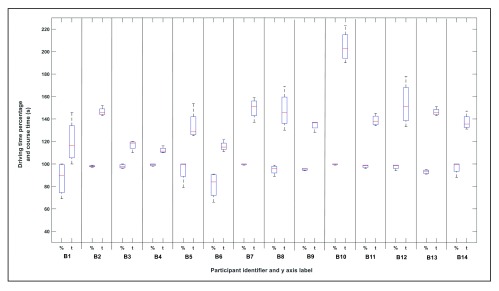
Course time and driving time percentage for all fixed course participants.

The driving ratio is given in the left hand column of each participant and the total course time in the right hand column in Figure 19. The large range of both ratio and overall time for B1/C1 was thought to be because the participant tried to concentrate too hard on not making a mistake rather than undertaking the course as quickly as possible. Participants B6 and B7 were novices and therefore unfamiliar with PWC’s response to their input. This caused their stop/drive ratio and overall time to be irregularly varied. Participants B7, B8, B10, and B12 had a narrow driving to stop ratio range but a varied overall time to complete course range which might have suggested tiredness or cognitive difficulty, participants reported these issues at the end of the test; however there was no direct correlation to each run.

It is clear when looking at the three day data that without determining exactly what task the PWC was undertaking, such as a fixed course, the ratio and overall PWC use time can only be a long term or day-to-day feature rather than over each day. However, a smart assistive PWC system would potentially be able to identify the platform location and therefore determine, for example, that the user was taking too long to negotiate the bathroom doorway and use that information together with the obstacle proximity feature to determine that the user is in need of greater assistance.

### Pattern recognition analysis of feature set

The purpose of this research is to identify potential features and metrics that identify user driving patterns and changes in those patterns over time. This information can then be used so that assistive PWC systems can adapt over time to the user’s changing needs. Therefore the features must indicate that changing state. In order to test this we have used classifiers which can be used to run in real-time on embedded hardware. One task is to examine whether the driving characteristics enable the identification of each participant.

Whilst there are not many samples, they can be reasonably divided into testing and training sets ( Table 15) with ten tests run for each classifier to improve robustness of testing. The outcome determined that it was possible to identify each participant between 74% and 86% correctly ( Table 16) despite only having a limited dataset. If the richer dataset for group ‘C’ is used then, as can be seen in Table 17, there was a certainty of correct identification between 86% and 95%, dependent on the classifier used, between C1 and C2.

**Table 15.** Pattern recognition testing criteria.

Test	Total samples	Classes	Training/ test split
All participants test	56	14 (users)	50:50
A versus B test	140	2 (A and B)	60:40
A change over time test	35	3 (Day 1, 2 ,3)	60:40
B change over time test	105	3 (Day 1, 2 ,3)	60:40

**Table 16.** Pattern recognition test of driving test course participants.

	All participants test number										
Classifier	1	2	3	4	5	6	7	8	9	10	Ave
**Linear Bayes** **Normal Classifier**	89.3	82.1	89.3	89.3	89.3	82.1	78.6	89.3	85.7	85.7	86.1
**K-Nearest** **Neighbour-1**	89.3	82.1	75.0	82.1	82.1	85.7	89.3	75.0	85.7	75.0	82.1
**Naive Bayes** **classifier**	82.1	78.6	82.1	92.9	85.7	75.0	78.6	71.4	82.1	89.3	81.8
**Parzen classifier**	92.9	85.7	85.7	89.3	85.7	82.1	78.6	85.7	78.6	82.1	84.6
**Fisher’s linear** **Discriminant**	71.4	75.0	71.4	67.9	89.3	75.0	75.0	71.4	71.4	75.0	74.3
**Logistic linear** **classifier**	83.9	75.0	78.6	85.7	80.4	73.2	78.6	78.6	89.3	85.7	80.9
**Support vector** **machine**	82.1	89.3	67.9	82.1	64.3	78.6	78.6	75.0	85.7	75.0	77.9

**Table 17.** Pattern recognition test of three day participants for individuality.

	C1 versus C2 test number										
**Classifier**	**1**	**2**	**3**	**4**	**5**	**6**	**7**	**8**	**9**	**10**	**Ave**
**Linear Bayes Normal** **Classifier**	91.8	90.0	90.0	95.4	89.1	91.2	93.6	94.5	90.9	90.9	91.7
**K-Nearest Neighbour-1**	96.4	90.9	98.2	92.7	96.4	96.4	92.7	87.3	98.2	98.2	94.7
**Naive Bayes classifier**	83.6	80.1	87.3	90.9	89.1	85.4	85.4	88.2	90.1	81.2	86.1
**Parzen classifier**	90.1	92.7	92.7	96.4	96.4	100	92.7	92.7	94.5	98.2	94.6
**Fisher’s linear** **Discriminant**	92.7	88.2	90.0	95.4	88.2	93.6	93.6	90.0	88.2	91.2	91.1
**Logistic linear** **classifier**	90.0	89.1	93.6	91.8	90.0	92.7	90.0	91.8	88.2	88.2	90.5
**Support vector** **machine**	92.7	94.5	91.8	91.8	86.4	90.0	90.9	92.7	90.9	94.5	91.6

When data from C1 and C2 were analysed as it changed over the three days ( Table 18 and Table 19), by labelling each day and comparing the three days for each participant individually, there was a much lower correlation of 65% to 85%, indicating that there had been a variation between the days. There was of course variation during each of the days and this was not differentiated by the labelling due to lack of ground truths and specific tasks undertaken over the course of each day, this requires further investigation in a more observed environment. However, these results clearly show that there is already potential for adjusting the system over the long term without the ground truths, which is an important step towards developing an automated assistive system.

**Table 18.** Pattern recognition test for changes over the three day period (C1).

	C1 change over time test number										
**Classifier**	**1**	**2**	**3**	**4**	**5**	**6**	**7**	**8**	**9**	**10**	**Ave**
**Linear Bayes Normal** **Classifier**	66.7	63.0	51.9	77.8	74.1	63.0	77.8	51.9	63.0	59.3	64.9
**K-Nearest Neighbour-1**	70.4	70.4	85.2	63.0	70.4	77.8	85.2	77.8	55.6	77.8	73.4
**Naive Bayes classifier**	88.9	74.1	96.3	81.5	92.6	85.2	85.2	88.9	74.1	85.2	85.2
**Parzen classifier**	63.0	70.4	66.7	59.2	70.3	59.2	66.7	55.6	85.2	66.7	66.3
**Fisher’s linear** **Discriminant**	48.1	48.1	66.7	66.7	63.0	63.0	70.3	66.7	77.7	81.5	65.2
**Logistic linear** **classifier**	70.3	85.2	62.9	55.6	85.2	81.4	85.2	63.0	63.0	85.2	73.7
**Support vector** **machine**	66.7	70.4	55.6	66.7	81.5	59.3	77.8	85.2	77.8	74.1	71.5

**Table 19.** Pattern recognition test for changes over the three day period (C2).

	C2 change over time test number										
Classifier	1	2	3	4	5	6	7	8	9	10	Ave
**Linear Bayes Normal** **Classifier**	71.6	64.2	59.3	74.1	72.8	69.1	64.2	76.5	72.8	66.7	69.1
**K-Nearest Neighbour-1**	82.7	77.8	70.4	85.2	77.8	82.7	90.1	85.2	82.7	87.7	82.2
**Naive Bayes classifier**	74.1	75.3	69.1	71.6	69.1	75.3	80.2	74.1	69.1	79.0	73.7
**Parzen classifier**	76.5	79.0	83.9	71.6	87.7	77.8	82.7	71.6	74.1	72.8	77.8
**Fisher’s linear** **Discriminant**	74.1	76.5	65.4	71.6	69.1	72.8	71.6	72.8	71.6	60.5	70.6
**Logistic linear** **classifier**	71.6	64.2	76.5	71.6	71.6	77.8	70.4	76.5	74.1	72.8	72.7
**Support vector** **machine**	69.1	75.3	69.1	75.3	77.8	71.6	75.3	70.4	74.1	65.4	72.3

## Conclusions

The development and provision of effective assistive technology to enable an individual to perform daily tasks on a more equal basis to someone who does not have the same disability can be challenging and hard to quantify, let alone justify, when funding demands are forever stretched. This research has sought to ease the development of an adaptable adjustable system by identifying some quantitative and qualitative measures with which to test the requirement of providing adaptive assistance to the PWC user in addition to determining the driving features and adjustable elements.

It is interesting to note from the results that, when assessing individuals, their awareness of the circumstances, location, and level of observation significantly affects their behaviour and performance. Therefore information as to the user’s location and the task they are undertaking needs to be additionally identified by the smart system for it to be able to provide a robust adaptive system.

This research has identified that:

- The mapping of Joystick position to speed and turn interpretation can be adjusted over the long term as the user‘s movement pattern changes. An initial setup mapping can be undertaken for calibration and recalibrated sometime later. From the results, it does not appear to be necessary to update over the very short term such as during the course of a single day. Without knowing what the time related task being undertaken, there was no meaning to the short term time duration as we discovered when we undertook the long term 3 day testing. For example, there was no distinction between the users just moving the joystick accidentally or out of boredom and actual intended motion. The time feature has a clear function to be employed as a measure of how quickly a user can undertake a specific set task, this translates to the passing through of doorways and passageways where they are identifiable by the system. The time function can also indicate the general trend of usage over time. The ratio of driving time can be used as a long term trend potentially indicating the user’s ability change over time. This feature can also be used on task specific activities as a measure of the user’s quality of navigation and therefore is a factor in the user abilities as they change over time during the day and over the days which can also indicate the need for a change in the level of required assistance.
- The smoothness feature is good for identifying the long term trend, when combined with tremor frequency, and as a short term daily identifier of the need to filter the input signal. This feature also has the important characteristic of being an identifier for the need to change the level of assistance from a smart assistive PWC.
- The frequency of tremor obtained from the joystick is a good measure of user change over time providing the time frame is of sufficient length to discount short term anomalies caused by controlled user actions which mimic a tremor. The smoothness feature should be used for the short term input filtering.
- The sensor feedback from the collision avoidance system can be used to indicate the proximity of obstacles as the user negotiates the environment. This produces a feature when the user input is compared to the system generated output that can be used to adjust the assistance, and level of assistance offered by the system.
- Finally the velocity vector can be used to determine the level of user uncertainty at some moment in time, such as when negotiating tight spaces and the user over reacts or makes erratic corrections. This is an interesting and important observation.

The paper has therefore identified features and metrics which, with further refinement and testing, are suitable to be used to set up industry standard PWC control systems and to monitor their use for adjustment as the user’s needs change over time. This is a significant improvement over the current trial and error method and these features and metrics can be used to adjust/correct user joystick input to keep them safely in control of their machine for longer rather than crossing some digital threshold and denying them control. Further work is required to obtain user ground truths with respect to user actions, and to monitor their changes in behaviour over longer periods of time, when participants are using their own PWCs in order to determine precise adjustments for the smart adaptive system. This would need to involve accurate observation of panicky movements and spasms and users negotiating real-world obstacles.

## Data availability

The data supporting the findings reported in this study have been uploaded to OSF: https://osf.io/w95ba/ ^42^. The following files are included:

### Extended_Day_Data.zip

This folder contains all of the data collected over the extended experiment which includes: Time in milliseconds; joystick speed; joystick turn; drive profile; x body accelerometer; y body accelerometer; z body accelerometer; body roll; body pitch; body yaw; and collision event. Only days which contained sufficient data were reported in the results.

### Fixed_Course_Feature_Results.xlsx

This file lists the features identified for each participant on all four attempts of the driving course.

### Initial_Joystick_Data.zip

This folder contains the artificially generated tremors for determination of the suitability of the PWC joystick data rate from the manufacturers system CAN Bus.

### Joystick tremor analysis.xlsx

This file summarises the artificially generated joystick tremors.

### Pattern_Recognition_Combined_Data.zip

A folder containing the feature data for the pattern recognition testing from the driving course and the multiple day usage.

### SANAS_Driver_Symptoms_Data.xlsx

Anonymous questionnaire responses.

### Wheelchair Fixed course Data.zip

Anonymized data from the 14 participants on the driving course assessment with key.

## Supplementary material

Supplementary File S1: Questionnaire for the collection of data from powered wheelchair users.
