# Supplementary material for: Feature determination from powered wheelchair user joystick input characteristics for adapting driving assistance
Source: Wellcome Open Res. 2018 May 10;2:93. Originally published 2017 Sep 27. [Version 3] doi: 10.12688/wellcomeopenres.12280.3 (PMC5829523; doi:10.12688/wellcomeopenres.12280.3)
Supplement: Supplementary file 1 [file wellcomeopenres-2-15903-s0000.tgz › 2783f7aa-5572-4455-bcdd-7104b8071d78.pdf]

University of Kent | School of Engineering and Digital Arts

SANAS

A synergetic adaptive non-intrusive-navigation assistance system for empowering the disabled, elderly and infirm powered wheelchair user

Powered wheelchair user data collection

April 2016

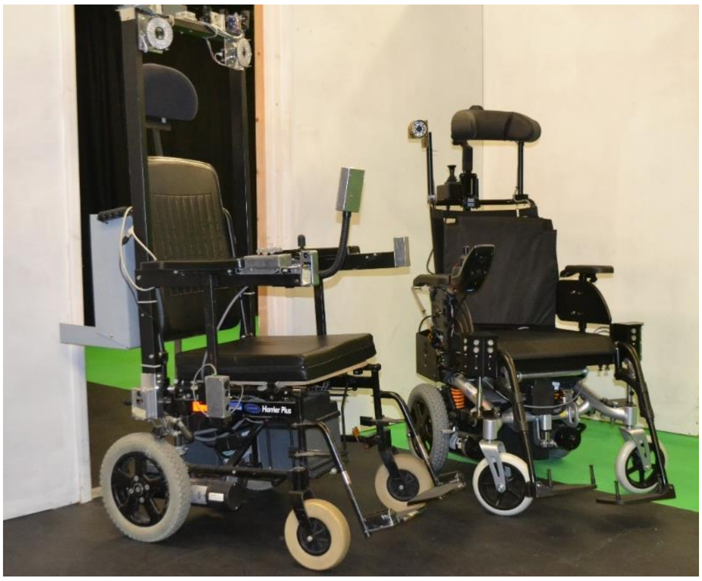

Supported by wellcome trust

We would like to thank you for volunteering to participate in our research to develop an adjustable assistive navigation aid for safer driving of a powered wheelchair (PWC). Our aim is to provide assistance, not to take control away from the user and we need you to help us determine the range of assistance that is required.

This project follows on from previous smart PWC developments, during which we identified the need to develop a smart and adaptive system which is suitable for assisting users, who have a wide range of medical conditions, and assistive requirements, that may vary over time. The user joystick input quality is a measure of the level of assistance required. However little research has been undertaken to quantify and qualify user inputs with regard to providing specific assistance. For example, one user may find turning left problematic. To provide automated assistance, the quality of the user input may be consulted as it would be highly likely to identify the user difficulty. The navigational assistance would then be automatically adjusted to provide more help when turning left. Furthermore, recognising the characteristic input patterns could be used to give an indication for the change in a user’s medical condition as the user’s ability to use the system may vary over time.

An adaptive system would allow the user to negotiate obstacles with far greater flexibility using a more natural and consistent trajectory, which is independent of the user’s fluctuating physical abilities. If the user requires specific assistance then the system would recognise this and provide the necessary correction to the user trajectory. In all situations, the user remains fully in control of the stop-go and velocity input; the system will uniquely damp the inputs, not increase them, making the system safe. The assistive trajectories we have developed have been experimentally evaluated. The testing indicates they correctly align the wheelchair platform for collision-free doorway passing from various incorrect approach angles and translations.

We would like you to undertake a recording of your PWC driving on two typically active days; one when you are at your best ability and the other when you are at your worst ability, where your ability is judged as both physical and mental. Both days should ideally contain the same activities and time frames in order to fairly compare the two.

The recording forms are included in this pack as well as an envelope to return them to us when you are finished. When completing the forms you should:

- be consistent when recording information
- attempt to complete the form fully
- not make a random guess if you are not sure, leave it blank
- not worry if you miss something, leave it blank
- not allow the recording of the data to affect your well being
- know that we will not use any personal information

Contact:

Dr Michael Gillham: M.Gillham@kent.ac.uk

Dr Gareth Howells: W.G.J.Howells@kent.ac.uk

The University of Kent
School of Engineering and Digital Arts
Jennison Building
Canterbury
Kent CT2 7NT

How many years have you been using a manual wheelchair?	
How many years have you been using a powered wheelchair?	

Why do you use a powered wheelchair (PWC)?

____

____

What makes the PWC difficult for you to use/control?

____

____

How would you rate your driving ability from 1 to 5? (Where: 1 = novice ……. 5 = expert)	

Please complete and return to us with the activity sheets.

Please complete the **'symptoms'** table on page 3 by putting a number between 1 and 5 in each of the boxes on the right for the symptoms you suffer with, where:

1 = minor inconvenience

2 = causes occasional problems

3 = problematic effecting day-to-day tasks

4 = severely affecting personal performance

5 = unable to function without assistance

Please insert the values such that the lower range value means the least amount you suffer from these symptoms and the upper range the most you suffer with on a daily basis.

For example; if you suffer with concentration difficulties, this might range from not being able to read newspapers on a 'good day' to not being able to communicate with people on a 'bad day'. You might then put the 'good day' for code '**B**' as '**1**' and the 'bad day' as '**5**' as in the example shown below.

**B**	Attention and/or concentration difficulty	1	5

Code	Symptoms	Good day	Bad day
**A**	Muscular tremors and/or spasms		
**B**	Attention and/or concentration difficulty		
**C**	Panic and/or agitation (nervousness)		
**D**	Reasoning and/or confusion		
**E**	Muscular stiffness		
**F**	Muscular weakness		
**G**	General fatigue/tiredness		
**H**	Observational and/or visual bias		

Please complete and return to us with the activity sheets.

We would like you to record the total number of collisions that you may have with specific obstacles, which are listed in the ‘activity sheet’. In the columns marked accidental collisions, deliberate collisions, and direction changes please record the number of times that these occur for the entire duration of the day and note for how long you operated the chair over the course of the day. **Please record one sheet for one of your ‘good days’ and one for one of your ‘bad days’.**

An accidental collision is when you damage, or scratch, or mark the object (not gently touch the object) without meaning to do so.

A deliberate collision is when you need to push the door open with the chair or there is not enough space to pass through without the chair touching something, in other words these collisions cannot be prevented.

Direction changes are occasions when you have aligned or positioned the chair incorrectly and have had to reverse the chair backwards and tried to approach again or have had to go another way; direction changes should also be recorded if you got confused or forgot which way to go and then went the wrong way.

Please use spare copies of the activity sheet to mark the events as they occur in a manner like that shown below. When recording the amount of time you used your chair, for the time divisions you could make a mark every ten minutes of use.

**Type of day**	~~Good~~	Bad
**Total amount of time you used your PWC during the day in minutes**		|||||||||||||||

**Obstacles**	**Accidental collisions**	**Deliberate collisions**	**Direction changes**
Door	||||||||	|||||||||||	
Doorway frame	|||		|||

……..Etc.

Then when you have finished collecting the data please transfer the totals to another activity sheet, like shown below, and return your 'good day' and 'bad day' records to us in the envelope provided together with pages 1 and 3.

**Type of day**	~~Good~~	Bad
**Total amount of time you used your PWC during the day in minutes**		130

**Obstacles**	**Accidental collisions**	**Deliberate collisions**	**Direction changes**
Door	7	10	0
Doorway frame	3	0	3

……..Etc.

Activity sheet

Type of day	Good	Bad
**Total amount of time you used your PWC during the day hours and minutes**		

Obstacles	Accidental collisions	Deliberate collisions	Direction changes
Door			
Doorway frame			
Walls			
Furniture			
People or animals			
Road surface			

Please complete and return two of these to us.

Activity sheet

**Type of day**		Good	Bad
**Total amount of time you used your PWC during the day hours and minutes**			

**Obstacles**	**Accidental collisions**	**Deliberate collisions**	**Direction changes**
Door			
Doorway frame			
Walls			
Furniture			
People or animals			
Road surface			

Please complete and return two of these to us.
