# Supplementary material for: Feature determination from powered wheelchair user joystick input characteristics for adapting driving assistance
Source: Wellcome Open Res. 2018 May 10;2:93. Originally published 2017 Sep 27. [Version 3] doi: 10.12688/wellcomeopenres.12280.3 (PMC5829523; doi:10.12688/wellcomeopenres.12280.3)
Supplement: Peer review history

# Peer review: Referee response for version 3

**DOI:** 10.21956/wellcomeopenres.15903.r33126

**Published:** 2018-05-14

**Authors:** Lorraine H DeSouza

**Affiliations:** 1 The Institute of Environment, Health and Societies, Brunel University London, Uxbridge, UK

Appropriate amendments have been made

I have read this submission. I believe that I have an appropriate level of expertise to confirm that it is of an acceptable scientific standard.

# Peer review: Referee response for version 2

**DOI:** 10.21956/wellcomeopenres.14387.r28750

**Published:** 2018-02-27

**Authors:** Juan Carlos García

**Affiliations:** 1 Department of Electronics, University of Alcala, Alcalá de Henares, Spain

Most of the issues pointed out in my first review have been addressed successfully. Some of them were clarified with the answer given by the authors to the first review.

 Nevertheless the content of section “Proximity to obstacles” can be improved in the same way I pointed out the first time. Equation 9 is a ‘dark formula’ with unknown parameters and effects to readers of this paper. The reader must access to reference 14 in order to get the definitions and effects of the function F defined on equation 9. So, this revised version of the paper still have a lack of details in this section. It is not required a full definition of all the parameters across this section but, in its present form, concepts the parameters ‘R’, ‘p’ and ‘k’ as well as the actual effect of ‘F’ are still useless for readers.

 Also, the function F can be confused with the name ‘F’ assigned to the secondary focus (F, as well) of the wheelchair surrounding area, as defined in Figure 4. After reading reference [14] it seems that function ‘F’ (equation 9) is the damping factor that is drawn in figure 4. But the distance to obstacles depends on the definition (not given here) for parameters R and p.

 Many of the equations shown here are (or can be) of general knowledge for readers, like the he wheelchair dynamics, eqs. (1) and (2), and the FFT definitions on eqs. (3) and (4). Equations (5) to (7) have enough details for a first reading of this paper. But equations (8) and (9) have almost no details nor parameters’ definition. The quality of this paper will be improved when such issues were fixed.

 Another issue that can be also revised is the two upper plots on Figure 6. If I am right, both contains the same data but the second plot also includes the peak detection results. Under my point of view the peak detection performance can be checked better joining both plots and make a single one but with a wider vertical scale.

 I have to congratulate the authors because the big amount of data provided within this research and the presentation of detailed statistics and tables. I hope that such a detailed data sets could help to create suitable joystick behaviour on-line identifiers.

I have read this submission. I believe that I have an appropriate level of expertise to confirm that it is of an acceptable scientific standard.

# Response

**Published:** 2018-05-03

**Authors:** Michael Gillham

**Affiliations:** University of Kent, UK

The authors would like to thank the reviewer for helping to improve the quality of the paper. We decided to leave figure 6 as it is, because it is used to explain the process rather than to present the data i.e. the raw data from the joystick can be either analysed using the peaks method or the Fourier analysis.

# Peer review: Referee response for version 1

**DOI:** 10.21956/wellcomeopenres.13294.r26860

**Published:** 2017-10-26

**Authors:** Lorraine H DeSouza

**Affiliations:** 1 The Institute of Environment, Health and Societies, Brunel University London, Uxbridge, UK

In the Introduction, the statement “Young people’s needs, with regards to the benefits of the PWC, have not been fully researched” relies on ref 5 which is now 14 years out of date. The authors need to carry out a contemporary literature search and review up-to-date research studies. This needs attention and the findings from a search may further inform or adjust the evidence underpinning the research aims and objectives.

 The “ability-to-operate test” is carried out to assess individuals for PWC provision is primarily concerned with safety (chair user and others in the environment) and has more to do with how the chair user perceives, judges and interprets the environment in order to drive the PWC successfully.

 It is unclear from Table 1 what are the disabilities “which currently prevent individuals from operating a PWC without assistance”. Many of those listed e.g. spasticity, weakness, would not prevent use of a PWC. Some listed are co-occurring e.g., spasticity/hemiplegia. This needs clarification. 

 The statement that “This process may take several sessions and sometimes a satisfactory outcome is not achieved” requires the support of evidence specifically relating to the issues addressed in the paragraph, i.e. users’ control of the joystick.

 “Some of the observations commonly reported”. Please provide the sources of the reported observations that are considered to be common.

 What is the rational for including people who did not use powered chairs in group B? They are noted as ‘control’ – what does that mean? Is it that they did not have a health condition that would need a PWC for mobility? Also, what is the meaning of not applicable (N/A) for B12’s reason for having a PWC? The text states that there were 12 group B participants but table 4 indicates that there were 12. These issues need clarification.

 How were the activities of group C in the PWC recorded? Then, how were the recordings analysed? This needs to be addressed so that similar studies can reproduce the methods.

 Regarding the assessment of driving time and stationary time it would be helpful to reconsider the assumption that stationary time may “indicate their current capability” and “have some relationship to levels of tiredness and reasoning and thus some direct relationship to the amount of assistance which is required.” PWC users who work may spend a fair amount of time stationary (e.g. at a work station or operating a computer) and/or not driving the chair (e.g. because they may be driving their car/van/transporter travelling to and from work).

 How did the authors define a ‘good day’ and a ‘bad day’? How was this judged by the PWC users? Please provide a definitions. Table 5 and text – there are three users with high collision numbers – the majority in your category A. The remaining users had far fewer collisions. Is it fair to take an average? Similar comments are relevant for table 6. The same users A3 and A5 have high numbers. Interestingly, A 10 has fewer collisions on a ‘bad’ day than on a ‘good’ day. Any particular reason noted? Also, did the report of collision with people allow differentiation between the PWC user colliding with another person and another person colliding with the PWC?

 Page 19. The authors state “due to physical and/or cognitive damage”. Please change ‘damage’ to ‘impairment’. The features of positional pattern and biases could also be due to posture. It is not clear if the participants were seated with postural support, or had sufficient trunk muscle ability to be independent in postural support.

 Page 27 para 2: please rephrase “ …on not making a mistake and a colliding rather than having undertaken the course …” as it does not scan well. Similarly “..use the PWC and unfamiliar with its response …” (were unfamiliar?)

 p.27 – this is an interesting scenario – “the user was taking too long to negotiate the bathroom doorway ..” and so needed assistance. Whilst it is clear that the proximity device will sense an obstacle, would it also sense water spillage on the bathroom floor? This would be a safety reason for the PWC user to stop at the doorway.

 Typo – “When the data from C1 and C2 was analysed”  data *were* analysed.

 The authors have appropriately summarised what this research has identified. They should also discuss the limitations of the study. One issue arises that it would be helpful to have the views of the researchers about. The test circuit used had fixed features, yet everyday outdoors driving for PWC users requires manoeuvring and negotiating moving features – e.g. pedestrians, cars/bicycles etc, trolleys, buggies, etc. how would the system work in these everyday circumstances? Although the authors did not research this in the study, is it something that will need specific further research in order for the new devices to have benefits for users in daily life with a PWC?

 This review has not commented on the bioengineering aspects of the paper as the reviewer is not a bioengineer. A review by a peer bioengineer working in powered wheelchair research is recommended.

I have read this submission. I believe that I have an appropriate level of expertise to confirm that it is of an acceptable scientific standard.

# Response

**Published:** 2017-12-05

**Authors:** Michael Gillham

**Affiliations:** University of Kent, UK

We would like to thank the reviewer for the helpful and thorough review. We have subsequently made changes to the content, as suggested, with regard to all of the points raised.

# Peer review: Referee response for version 1

**DOI:** 10.21956/wellcomeopenres.13294.r26456

**Published:** 2017-10-16

**Authors:** Juan Carlos García

**Affiliations:** 1 Department of Electronics, University of Alcala, Alcalá de Henares, Spain

This paper is very interesting for future developments in Assistive devices for Powered Wheelchairs (PWC). However I have found several points that need to be clarified, justified or extended.

 The authors mention the “smart PWC collision avoidance system” (page 7, second paragraph) but they give no details about which is the smart PWC used. This information must be included here in order to know the actual expected performance of that avoidance system. If the set of users (A, B and C) had different PWCs such information should also be included, because this is an important factor that could affect the overall system’s performance.

 In the same page 7, Table 3, the concept “Driving ability” was included with an apparent numeric scale from 1 to 5. What does it mean? How was that figure obtained? Was it an objective quantity or subjective quality?

 The evaluation course shown in Figure 2 needs further details. There is no metric information there and the dimensions of a test driving course have a great importance when evaluating driving abilities. Full details about lengths and widths of corridors and dimensions and location of obstacles must be included.

 The section “Feature development” will be clarified if a list of such features is introduced before them, for instance after the first paragraph on page 9, just before the first feature studied (that is “input tremor and smoothness”). Also I guess that there is a miss-reference in the first paragraph on page 9: it is mentioned “Table 1” and I think that the right reference should be “Table 2”.

 On section “Joystick positional bias”, paragraph 2, there is a mention to axes x and y in Figure 3. Such axes were not represented there. Apparently they are supposed to be aligned with the Forward-Reverse (axis x) and the rotation (axis y) commands. Please: let it be clear on Figure 3 and in text. I have to say that normally I expect to see Joystick actions as they are physically found on the actual device that is “Forward” action pointing upwards.

 The “Proximity to obstacles” section needs to be improved. Although authors make a direct mention to reference 14, in the current article Equation (9) has all of its parameters undefined so it is meaningless here. For instance, what is the “damping factor” mentioned in text? Please, fix that issue.

 Figure 4 shows the so called “collision avoidance zoning”. Also here appear a lot of undescribed features. Why the zones appear inside some elliptic regions? Why there are two (apparently) important points here? One of such points is the turning centre of the PWC (between the traction wheels, back on chair) but, what is the meaning of the other point of interest? What is the meaning of the small rectangles? Are they a representation of the PWC wheels? Why are there two elliptic regions?

 In section “Feature evaluation…” on page 12 appears up to 7 classifiers. But, are the input vectors the same for all the seven classifiers? Please, give more details about what features are used for each classifier and, if all of them are used, give a list summarizing them.

 In my opinion, the section “Joystick tremor and smoothness analysis” must be suppressed or deeply reformatted. Let us go directly to Figure 7. From there it is stated that the 50Hz digital info is enough for identifying tremor so no additional hardware is required. That is evident knowing the expected frequency characteristics of tremor and the 50Hz sampling frequency of the system bus. Such 50Hz frequency on system bus, according to sampling theorems, are enough for identifying frequency patterns of even 10Hz (theoretically, up to 25Hz). So there is no need for introducing new devices for up-sampling low frequency information (like the one of tremor) up to 250Hz. So the results on Figure 7 are self-evident and any difference there could only be derived from systematic errors on the measuring process.

 In Figure 10, the vertical scale is “Frequency” and “Smoothness”. However, the data columns were referred to as “T” and “S”. I think that “T” must be replaced by “F” of Frequency.

 About the section “Pattern recognition…” on page 27 there are some points that need further improvement. As I said before, somewhere the reader needs to find what were the actual features introduced to the classifiers. But here we need to know also the ‘output classes’. Such info is shown in Table 15, but it is not clear to me the meaning of all the classes. For instance: first row in Table 15 show 14 classes that can be identified as one per user in Table 4. But, what about the other classes in other rows? And also, why there is not an option for a “not classified” or “not identified” class?

 I hope my questions and comments could help to improve this article. Nevertheless I would like to congratulate the authors for their excellent work.

I have read this submission. I believe that I have an appropriate level of expertise to confirm that it is of an acceptable scientific standard, however I have significant reservations, as outlined above.

# Response

**Published:** 2017-12-05

**Authors:** Michael Gillham

**Affiliations:** University of Kent, UK

We would like to thank the reviewer for the helpful and thorough review. We have made amendments to the content as suggested by the reviewer to all of the points raised with the exception of: The joystick plot orientation of axes was intended to correspond to the collision avoidance (assistive technology) body frame of reference. Although the 50Hz data rate from the joystick is sufficient to determine tremor frequency up to 25Hz our intention was to determine whether velocity was an adequate replacement for jerk as a measure of smoothness (as reported by others). In this case the joystick measurement of velocity between two datum points is simply the rate of the actual distance travelled by the joystick; however jerk is fourth derivative of joystick position and therefore we needed to sample for jerk at a much higher rate in order to compare velocity derived smoothness with the Normalised Jerk Score. We were also looking to use large amplitude sudden velocity magnitude and direction changes as a measure of panic or sudden reaction to misjudging obstacles, these have a velocity/frequency much higher than normal hand tremors, and we aim to determine this in future work when ground truths are available. In addition all these changes would need to be monitored as they changed over short periods of time, such as negotiating a specific doorway where there may only be 50-100 samples whilst also over the general long-term monitoring and filtering of tremors and potentially spasms. Consequently in response to the review we have re-worded this section to make these things clearer because it did appear unnecessary.
